# Technology-Assisted Rehabilitation Gym for at-home Training (TARGeT) for stroke survivors: A study protocol for evaluating feasibility, safety, usability, clinical benefit, and costs

**DOI:** 10.1371/journal.pone.0355160

**Published:** 2026-09-02

**Authors:** Simone Kager, Hsiao-Ju Cheng, Christopher Wee Keong Kuah, Tengiz Samkharadze, Hao Ping Lin, Chwee Yin Ng, Tegan Kate Plunkett, Sam Lee, Niranjana Devi, Filzah Sumartono, Isaac Lu, Ananda Sidarta, Eloise Lie, Irina Hungerbuehler, Daryl Chong, Anoop Kumar Sinha, Chi Onn Tang, David Matchar, Giada Devittori, Christoph Kanzler, Asif Hussain, Bernardo Noronha, Muhammad Azhar, Gabriel Aguirre-Ollinger, Roger Gassert, Karen Sui Geok Chua, Nicole Wenderoth, Olivier Lambercy

**Affiliations:** 1 Future Health Technologies, Singapore-ETH Centre, Singapore, Singapore; 2 Department of Health Sciences and Technology, Rehabilitation Engineering Laboratory, ETH Zurich, Zurich, Switzerland; 3 Department of Computer Science and Artificial Intelligence, University of Technology Nuremberg, Nuremberg, Germany; 4 Institute of Rehabilitation Excellence (IREx), Tan Tock Seng Hospital Rehabilitation Centre, Singapore, Singapore; 5 Rehabilitation Research Institute of Singapore, Nanyang Technological University, Singapore, Singapore; 6 Department of Internal Medicine (General Internal Medicine) and Pathology, Duke University, Durham, North Carolina, United States of America; 7 Articares Pte Ltd, Singapore, Singapore; 8 Lee Kong Chian School of Medicine, Nanyang Technological University, Singapore, Singapore; 9 Yong Loo Lin School of Medicine, National University of Singapore, Singapore, Singapore; 10 Department of Health Sciences and Technology, Neural Control of Movement Laboratory, ETH Zurich, Zurich, Switzerland; University rehabilitation institute, SLOVENIA

## Abstract

Stroke is a leading cause of long-term disability, with more than half of survivors experiencing persistent upper limb impairments. Despite clear evidence that high-dose, repetitive, task-specific training is essential for motor recovery, many stroke survivors do not receive a sufficiently high therapy dose – particularly in the outpatient phase. Limited therapist availability, high care costs, and travel burdens remain key barriers to accessing rehabilitation training. While technology-assisted rehabilitation has emerged as a promising solution, most existing studies are limited to single-device interventions, are conducted in clinical settings, and/or require constant supervision, thereby restricting their scalability and impact. This study protocol presents the Technology-Assisted Rehabilitation Gym for at-home Training (TARGeT) trial (ClinicalTrials.gov: NCT06406569), which aims to evaluate the feasibility, safety, usability, clinical benefits, and economic potential of a novel, unsupervised, high-dose home-based therapy program that integrates multiple complementary technologies for upper limb stroke rehabilitation. In this single-centre, pilot feasibility trial, up to 30 subacute and chronic stroke survivors will complete six weeks of daily home-based therapy using three complementary rehabilitation technologies that enable self-administered training without therapist supervision. The training will be remotely monitored by clinicians via an online platform that reports data on the training progress. Assessments will be conducted at five timepoints, measuring clinical outcomes (e.g., FMA, ARAT), quality of life, healthcare utilization, and technologies’ perceived usability (SUS, RawTLX). The study will assess whether unsupervised, home-based multi-technology rehabilitation is feasible and safe, provides high usability and satisfying user experience, whether it yields clinical outcomes comparable to conventional therapy based on data from previous studies (own data and literature data), and determines the associated costs relative to conventional therapy. The TARGeT program addresses the critical gap in therapy access and intensity by delivering scalable, high-dose rehabilitation directly into patients’ homes. Results will inform future trials and support broader adoption of decentralized, technology-enabled stroke rehabilitation.

## Introduction

Stroke is a leading cause of long-term disability, with approximately 50% of stroke survivors suffering from upper limb impairments [1,2]. To diminish sensorimotor impairments, stroke survivors can undergo physical rehabilitation and maintenance programs in hospitals or rehabilitation centres. Current evidence in stroke rehabilitation emphasizes the need for repetitive and intensive task-specific upper-extremity training to facilitate motor relearning and neuroplasticity [3]. While physical therapy aims to decrease functional dependence, care costs and burden on caregivers, and provide a higher quality of life [4], there is growing evidence that the therapy dose received by stroke survivors along the continuum of care is insufficient. This is especially prevalent in the (later) outpatient phase [5–7].

Changing demographics in many industrialized countries will increase the number of patients requiring neurorehabilitation further over the next years. An increased number of patients leads to a rising demand for healthcare professionals and a high burden on healthcare costs. Providing access to adequate doses of rehabilitation poses a significant challenge due to linked manpower shortages and high associated costs. Technological solutions, such as rehabilitation robotics, hold the promise to complement traditional therapy in cost-effective ways, especially if used without constant supervision from trained personnel. Upper limb technology-assisted therapy can provide task-specific, intensive, and repetitive exercises with comparative results in terms of impairment reduction and functional improvement as conventional therapy [8]. Complementing traditional one-on-one treatment with a therapist by technology can help decrease manpower requirements and therein, costs since minimal or no supervision is required [9,10].

While most of these technologies typically focus on one specific segment of the body, in recent studies, it has been proposed to combine several technologies to set up a circuit or gym training in the clinic that allows for more holistic training as typically present in post-stroke technology-aided neurorehabilitation [e.g., 11–14]. Bustamante et al. [11] showed that stroke survivors who underwent circuit training with six different devices according to a pre-defined time per station yielded descriptively similar clinical gains as those who received time-matched conventional care (each n = 10). The circuit training also proved to be more cost-effective due to the reduced supervision ratio of the therapists of 1:6 compared to 1:1 in the standard care group. Similarly, a multicenter clinical trial compared the time-matched training with four robotic devices to conventional therapy and could achieve comparable results across the treatment groups regarding the Fugl-Meyer Assessment (FMA) score [12].

Besides the limited availability of trained therapists, the challenge of receiving a sufficiently high therapy dose further aggravates as stroke survivors progress in their rehabilitation journey. In Singapore, it has been shown that rehabilitation adherence falls to 20% after three months of stroke onset, 10% after six months, and 4% after one year, with travel inconveniences, stroke survivors’ wish not to burden caregivers and high cost being the main reported barriers [15]. Tapping the full potential of technology-aided solutions would include adapting treatment and selection protocols, technology and monitoring systems to be directly accessible in the homes of stroke survivors [16]. Home-based technology-aided rehabilitation immediately reduces traveling burdens and associated costs, and has the potential to lower therapy costs due to the reduced demand for clinical resources. Additionally, training at home can lessen caregiver involvement if technologies with high usability are used, and can, thus, be used without continuous supervision from therapists. It further allows to distribute the training over a day as per one’s convenience which might reduce fatigue and enhance compliance [17]. Aguirre-Ollinger et al. [18] showed in a recent study that technology-aided therapy at home could be more cost-effective than clinic-based conventional therapy. Similarly, several other studies revealed favorable economic outcomes for home-based technology-aided therapy [19,20], whereby the cost reduction was achieved by eliminating therapist supervision and minimizing travel expenses. Moreover, the first studies that employed single devices at stroke survivors’ homes for unsupervised therapy have shown that adherence to the therapy program is high and that functional outcomes could be improved, e.g., in [18,21–24]. Yet, these studies included only one device, hence, focused on one specific aspect of therapy. As a result, they may only be suitable for patients with certain levels of impairment and may not be adaptable to the changing needs of stroke survivors along their rehabilitation journey.

To address the increasing demand for comprehensive, high-dose therapy we aim to bring home-based technology-aided rehabilitation even further by combining the benefits of a robotic gym with home-based therapy. We propose to deploy three complementary rehabilitation technologies, each focusing on a different part of the upper limb, subsequently at stroke survivors’ homes. The technologies allow for comprehensive training, including sensory, motor, and cognitive aspects, while therapists can monitor the training progress remotely from the clinic. Specifically, these technologies are highly usable and allow for individualized therapy programs depending on the specific upper limb impairment and the changes therein over the course of rehabilitation (i.e., focus on proximal or distal parts), while drastically increasing upper limb practice intensity and duration compared to usual clinic frequencies while reducing the requirements for clinical manpower, caregiver, and travel expenses, leading to a potentially economically effective rehabilitation model.

In the following, we present the study protocol for the Technology-Assisted Rehabilitation Gym for at-home Training (TARGeT) feasibility study in which up to 30 subacute and chronic stroke survivors will undergo six weeks of unsupervised, self-administered upper limb therapy at home. Specifically, we designed this study protocol to i) investigate whether unsupervised, home-based therapy with a set of complementary rehabilitative technologies is feasible and safe for stroke survivors, ii) assess the usability and user experience of stroke survivors and therapists with respect to the proposed TARGeT program, iii) evaluate whether a clinical benefit comparable to conventional therapy as reported in [9] can be achieved, and iv) to perform an economic analysis with respect to conventional therapy with data of previous studies [18]. The outcomes of the study will inform whether the TARGeT program is a feasible, safe, and economical way to tackle the increasing demand for post-stroke upper limb neurorehabilitation.

## Methods and analysis

### Study design, setting and participants

**Fig 1:**
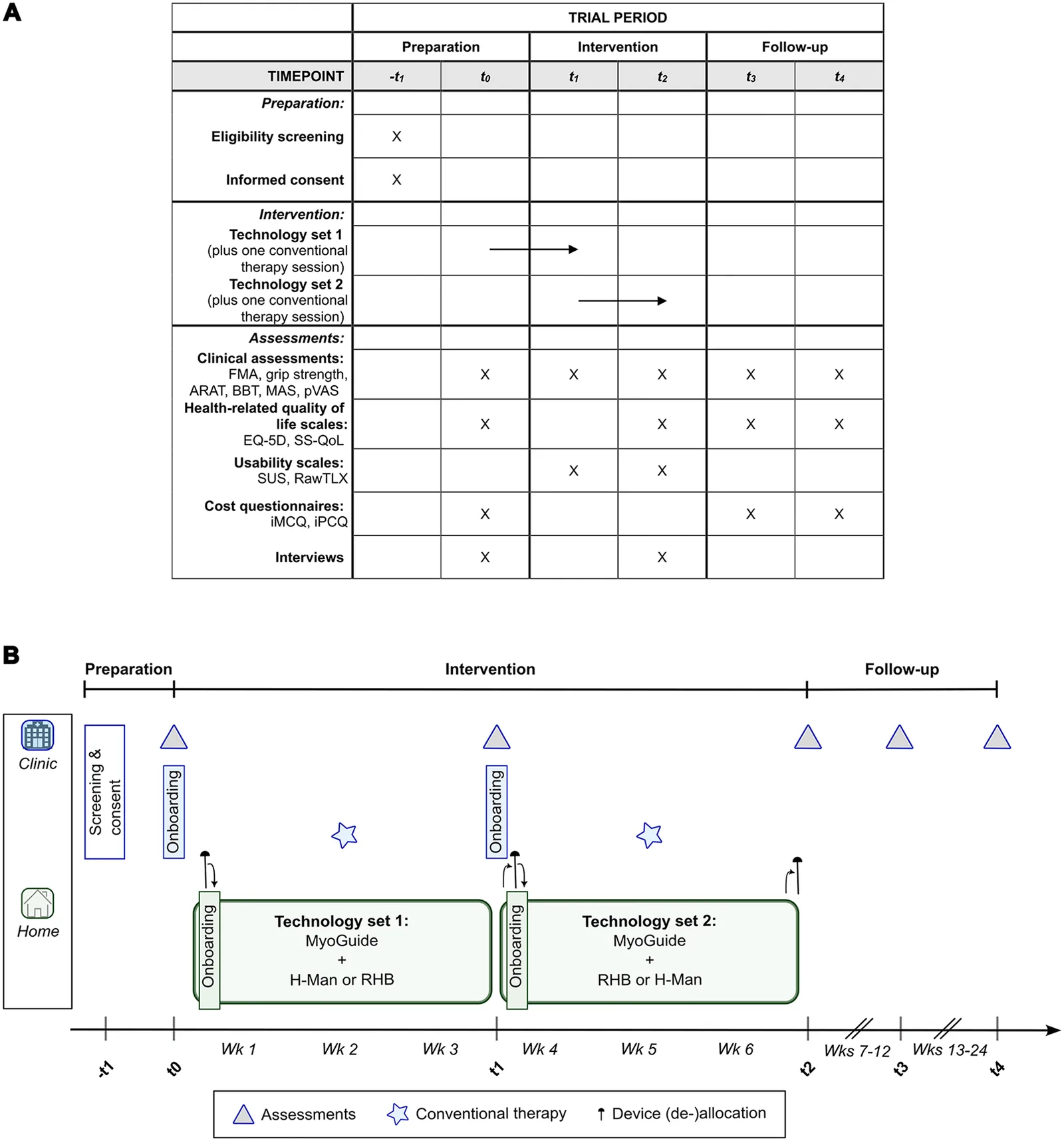
TARGeT intervention: Protocol and procedures. The protocol consists of a preparation phase in the hospital (assessments, device onboarding), a 6-week home-based intervention where participants train using the technologies, and a follow-up phase in the clinic to assess the effects of the intervention. A) Schematic participant timeline according to the SPIRIT reporting guidelines, B) illustrative protocol overview.

The study design is a clinical, single-group, open-label, pilot feasibility trial to evaluate the concept of a novel therapy concept, TARGeT program, in which three complementary neurorehabilitation technologies targeting different segments of the upper limb are deployed at stroke survivors’ homes in Singapore (Fig 1). Participants will undergo six weeks of intensive at-home therapy successively using these technologies according to tailored therapy plans while the training progress is remotely monitored by clinicians. The study will be conducted in the rehabilitation outpatient clinic of Tan Tock Seng Hospital, Clinic for Advanced Rehabilitation Therapeutics, National Healthcare Group (NHG) and in participants’ homes.

Ethical approvals were obtained from the NHG Domain Specific Review Boards on 13 June 2024 (ECOS reference number: 2023/00527, protocol version 2, see S2 File) and ETH Zurich Ethic Commission on 11 March 2024 (Reference number: 23 ETHICS-342). The study will be conducted according to the Declaration of Helsinki and all participants will provide signed written informed consent to the clinical team before participation. The study was registered on ClinicalTrials.gov with the following registration number: NCT06406569 on 06 May 2024. Additionally, for personal data protection and cybersecurity, approval from Singapore’s national health tech agency, Synapxe, was obtained before the commencement of the study. Results of the study will be made available in scientific publications.

This manuscript was written in accordance with the SPIRIT (Standard Protocol Items: Recommendations for Interventional Trials) guidelines [25] (see S1 File).

With reference to pilot studies, sample sizes in the range of 10–30 participants are recommended [26]. We will therefore recruit up to 30 participants, aiming for a sample size of 25, factoring in a drop-out rate of about 20% (5 participants).

The inclusion and exclusion criteria for participants were derived from experiences in previous studies with the technologies used here [9,18,27,28].

The inclusion criteria are as follows:

- Clinical stroke (ischaemic or haemorrhagic) confirmed by admitting doctors and brain imaging (computed tomography (CT), CT angiography or magnetic resonance imaging)
- Age 21–80 years
- At least 28 days post-stroke
- Upper limb motor impairment of FMA scale [29] 20–60
- Montreal Cognitive Assessment (MoCA) [30] > 21/30
- Ability to sit supported and continuously for 60 minutes
- Stable home abode with enough space to place technologies (clear floor area of at least 1.2m x 2.0m in the proximity of a power socket)
- Availability of caregiver to assist in home-based exercises

Participants will not be recruited if any of the following exclusion criteria are present:

- Functional impairment of the upper limb due to other pathologies than stroke
- Medical conditions incompatible with research participation: uncontrolled medical illnesses (hypertension or diabetes, ischaemic heart disease, congestive heart failure, bronchial asthma, severe/untreated depression, agitation, end-stage renal/liver/heart/lung failure, unresolved cancers)
- Anticipated life expectancy of less than six months
- Pacemakers and other active implants
- Active seizures within three months
- Local factors potentially worsened by intensive technology-aided upper-limb therapy and computer-based training:
  - Spasticity - modified Ashworth Scale (MAS) [31] > 2 of any upper limb muscle groups
  - Severe pain in affected arm - Visual Analogue Scale for pain (pVAS) [32] > 5/10
  - Skin wounds
- Severe visual impairment or visual neglect affecting the ability to use technologies
- History of dementia, depression, or behavioural problems
- Pregnant or lactating females

After recruitment, participants may be withdrawn from the study in the following circumstances:

- Poor fit to the technologies that could potentially cause complications (e.g., strain)
- Safety concerns (e.g., dizziness due to screen use)
- Serious adverse events related to the technology
- New medical problems unrelated to study intervention: recurrent stroke, new organ failure, new diagnoses rendering subject ineligible
- Not complying with the study procedures

### Rehabilitation technologies

To provide a comprehensive therapy program, three rehabilitation technologies were selected that complement each other by focusing on different segments of upper limb post-stroke therapy and that are easy to use in unsupervised settings which we consider as a key requirement for home therapy. From proximal to distal, H-Man focuses on sensorimotor functions of the shoulder and elbow, ReHandyBot on the forearm and hand, and MyoGuide on the wrist and hand. All technologies comprise multiple engaging exercises for user interaction and come with intelligent algorithms that adapt the challenge level of the interaction in the exercises based on the individual performance of a user to allow for unsupervised training. Moreover, all technologies have been previously successfully evaluated in terms of usability and/or clinical benefit for stroke survivors in separate pilot trials [9,27,28].

#### H-Man.

**Fig 2:**
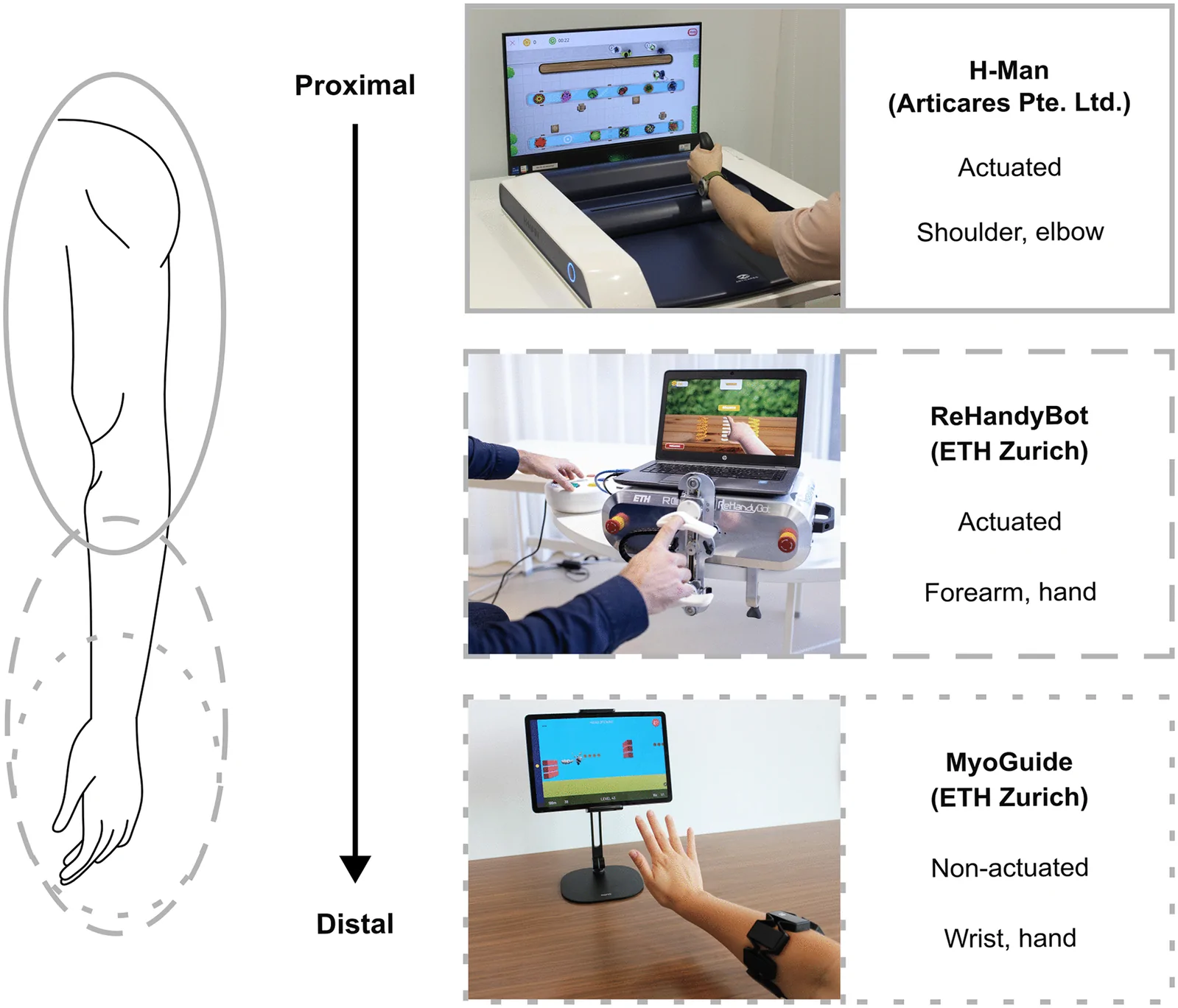
Technologies used in the TARGeT study. The three technologies employed at participants’ homes for daily training focus on different segments of the upper limb. From proximal to distal: H-Man on shoulder and elbow, ReHandyBot on forearm and hand, and MyoGuide on wrist and hand.

H-Man is a table-top end-effector robotic technology that allows planar movements targeting shoulder-elbow function. It was designed at Nanyang Technological University, Singapore [33] and is commercialized by Articares Pte. Ltd. (Fig 2). Through a gamified environment, H-Man provides repetitive, performance-adaptive exercises that target relearning sensorimotor control for resuming activities of daily living while also training cognitive functions. Depending on the participant’s needs, end-effectors with different grasp modes and an arm support for gravity compensation can be provided. H-Man has been successfully employed previously in neurorehabilitation therapy and assessment of sensorimotor functions in stroke survivors [34,35]. In a recent study, H-Man was successfully tested for unsupervised use in stroke survivors’ homes [18].

#### ReHandyBot.

ReHandyBot, which was developed by the Rehabilitation Engineering Laboratory at ETH Zurich, is a compact and portable two-degree-of-freedom end-effector robot that trains and assesses grasping movements, forearm pronation and supination [36] (Fig 2), and is a portable redesign of the ReHapticKnob [37]. It is embedded in a therapy platform proposing a set of exercises based on the neurocognitive therapy concept [38], to assess and train motor and sensory functions in the context of challenging tasks requiring cognitive processing (e.g., exploring and recognizing virtual objects with different mechanical properties rendered by the robot [39]). Exercises present different difficulty levels that are automatically adapted depending on participants’ impairment level (assessment-driven therapy) and performance during therapy [40,41]. ReHandyBot comes with a color-button keyboard that allows for easy navigation through the user interface. In previous studies with ReHandyBot and its predecessor in stroke survivors, it was shown that assessment-driven robot-assisted therapy leads to non-inferior recovery compared to dose-matched conventional care [27]. A recent feasibility study showed that minimally supervised therapy with the robot is possible and well accepted by stroke patients, with the technology showing high usability [10,24].

#### MyoGuide.

MyoGuide is a non-actuated technology for specifically training wrist extension and hand opening movements via biofeedback. Finger extension (hand opening) is a fundamental prerequisite for releasing objects and performing many activities of daily living [42], whereas sustained wrist extension provides stability for the hand during functional tasks and optimizes grasp force production [43–45]. It comprises an electromyography (EMG) armband (Myo armband, Thalmic Labs) for recording muscle activities from the forearm via eight EMG channels and a mobile device (e.g., tablet) to display the exercises and feedback [28]. The placement of the armband is not strictly defined but is recommended to be placed near the elbow to capture the extensors and flexors of both wrist and fingers. Our signal processing algorithm accommodates the armband placement and is able to derive reliable signals for game control. After user calibration tasks and technology-specific assessments, the user can engage in dynamic tasks targeting the control of wrist extension and hand opening (depending on the clinical impairment). The difficulty level of the exercise (i.e., the required muscle activation level, speed and accuracy of muscle control) is adapted based on the user’s performance. The usability of MyoGuide has recently been evaluated positively by subacute stroke survivors and their therapists [28].

### Protocol

All study participants will undergo a six-week unsupervised intervention with the described rehabilitation technologies at home (‘Intervention phase’), whereby they will train with two different sets of technologies, each for three weeks. The clinical progress will be assessed at five time points (Fig 1): pre (week 0, t0), mid (week 3, t1), post (week 6, t2), follow-up 1 (week 12, t3), and follow-up 2 (week 24, t4). The technology sets were selected as a combination of an actuated technology, i.e., rehabilitation robot H-Man or ReHandyBot, that allows for movements of the arm/hand with the user potentially remaining fully passive, and the non-actuated MyoGuide which trains muscle activation patterns. Hence, the first set includes MyoGuide plus H-Man or ReHandyBot, and the second set includes MyoGuide plus the respective other actuated technology that was not part of the first set. This design was motivated by the desire to provide participants with technologies supporting active (actuated) and passive (non-actuated) movement training throughout the intervention, but without requiring participants to have all technologies at home during the entire intervention. This would be suboptimal in terms of space requirements at home, and technologies availability for the study. Additionally, the combination of the technologies considers the space constraints typically present in houses in urban areas such as Singapore.

The Intervention phase will be preceded by a ‘Preparation phase’ and followed by a subsequent ‘Follow-up phase’ (Fig 1). Participants will be financially compensated for all study-related visits to the clinic.

#### Preparation phase in clinic.

This phase will consist of baseline assessments (t0) and two onboarding sessions for the first technology set (one for each device of the first set, e.g., MyoGuide and H-Man), conducted upon informed consent (-t1). The baseline assessment (t0) will include clinical scales, health-related quality of life (HrQoL) scales, and cost questionnaires. Details of the individual assessments are provided in Section 4.

Each onboarding session in clinic will last up to 120 minutes in which the participants will be trained in the operation of the technologies. As per advice from clinicians, participants might be jointly trained with their caregivers. The onboarding will follow a defined protocol inspired by our previous work [46] in which participants and caregivers will slowly transition from a fully supervised operation and training mode (i.e., the research therapist will instruct the participant on how to use the technology and closely observe the participant while training and interfere whenever needed), to a minimally-supervised training (i.e., the research therapist will only be available on demand).

After the second onboarding session, the clinical team will set up a therapy plan (60–90 minutes daily) for the unsupervised home training with the technologies of the first technology set. The therapy plan will allow for the automatic execution of the exercises, individually selected for the respective participant.

Onboarding sessions will not be considered as interventions with a therapeutic effect but serve to qualify participants and caregivers in the independent operation and interaction with the technologies in preparation for unsupervised training at home.

#### Intervention phase.

After the in-clinic onboarding, the first technology set will be installed at a participant’s home and a third onboarding session (up to 120 minutes) will be conducted at their home. In this session, the participant will be trained (together with the caregiver) on the safe and correct, unsupervised operation of and training with the technologies. If, after the third onboarding session, the participant and caregiver are not deemed proficient enough for unsupervised training, more onboarding sessions at home can be arranged. Also, if required, appropriate furniture for the interaction with the technologies will be provided. During the at-home onboarding visit, a semi-structured interview with the participant will be conducted to understand experiences and expectations with post-stroke rehabilitation.

The unsupervised training at home will then start with the first set of technologies for three weeks in which participants are instructed to train daily according to their therapy plan. The daily therapy plan comprises 15 minutes training with MyoGuide and 45 minutes using H-Man or ReHandyBot, with the option to extend the training to up to 120 minutes per day. This upper limit is based on clinical experience of the study team and is set to avoid excessive fatigue and injuries. It is also consistent with previous studies with unsupervised, technology-assisted therapy (e.g., [18]). The shorter duration of MyoGuide was chosen because it requires repeated, high-intensity muscle activations close to maximal EMG output, which can quickly lead to fatigue. The training can be split up into multiple sessions over the day as per the participant’s convenience. In the middle of this three-week training, participants will receive one add-on conventional therapy session in the clinic (Fig 1) to support the translation of the gained function. This session also offers the opportunity for the participants to discuss the training progress with the therapists. After completion of the three-week training with the first technology set, participants will return to the clinic for a fourth onboarding session with the remaining technology (e.g., if H-Man was part of the first set, they will be onboarded to ReHandyBot). Additionally, a mid-assessment (t1) will be performed, comprising clinical scales and usability questionnaires. Next, in an at-home onboarding session, the new actuated technology will be set up and the old will be collected. Again, the fitness for unsupervised training with the new technology will be assessed by the therapists and if needed additional onboarding sessions can be arranged. The participant will then be advised to train for another three weeks daily in an unsupervised manner with this second technology set according to the updated therapy plan (up to maximum 120 minutes daily). During this second part of the intervention, the participant will also return to the clinic for a conventional therapy session in the middle of the three weeks.

After the home training of six weeks, the study team will retrieve the technologies and conduct another semi-structured interview with the participant to understand their perception and experience with the unsupervised TARGeT program. The Intervention phase will terminate with a post-intervention assessment (t2) in the clinic to determine the immediate effects of the therapy including clinical assessments, usability evaluations, and HrQoL questionnaires.

In the beginning of the Intervention phase, i.e., few days after the onboarding session at home, therapists will call the participants to check on their ease of use and potential problems. During the remaining Intervention phase, therapists will remotely monitor the participants’ compliance with the therapy plan at least once per week (more details in Section 5) and will contact the participants via phone call or text message in case there has been no training with the technologies on the remote monitoring website for at least two consecutive days. Additionally, participants will be given the option to contact the therapists whenever needed. Any off-protocol contact required due to, e.g., non-training or technical issues of the technologies will be recorded in dedicated report forms for feasibility, safety, and economic analysis.

#### Follow-up phase.

The protocol will be finalized with the Follow-up phase, in which the long-term effects of the intervention will be assessed at six weeks (t3) and 18 weeks post-intervention (t4). The follow-up assessments comprise clinical assessments in the clinic, HrQoL scales, and cost questionnaires for the participants. Adverse events related to training will also be monitored at each follow up session, i.e. pVAS, MAS, skin issues etc.

### Outcome measures

Alongside the technology data that will be gathered with the rehabilitation technologies during the Intervention phase, and journal and report forms (primary outcomes), demographic, clinical scales, user experience, and cost data (secondary outcomes) will be collected at five time points (Fig 1).

#### Technology data.

The feasibility of the TARGeT program will be assessed based on adherence to the therapy plan, which is determined by both attendance and training dose. Attendance is defined as daily access to the technologies, measured through log-in and log-out times recorded by the technologies. The daily training dose is defined by the daily active training time, i.e., the duration spent performing the exercises, excluding planned breaks. For deeper future analyses, also, the exercises played, the aggregated number of task repetitions as well as the progression of the challenge levels per technology will be stored. Depending on the technology and the exercise, also the kinematic, dynamic, and EMG data as well as pre-established performance measures (e.g., smoothness [47]) will be saved.

#### Journal and report forms.

Stroke survivors, caregivers, therapists, and technical personnel will log the time spent on the intervention and report any interaction with the respective other parties that were not scheduled in the protocol in journal and report forms, e.g., identified by remote monitoring, as well as any safety and technical concerns encountered. From this data, the safety of the TARGeT program will be determined.

#### Clinical scales.

The clinical upper limb status of the participants will be determined at every time point (t0 to t4) with standardized clinical scales for motor impairment (FMA and Grip strength (GS) measured by a digital dynamometer), functional performance (Action Research Arm Test (ARAT) [48], Box-and-Block Test (BBT) [49]), spasticity of the shoulder adductors, elbow, wrist, and finger flexors (MAS), and pain (pVAS).

#### Usability scales.

To assess the usability of each technology, the System Usability Scale (SUS [50]) and the Raw Task Load Index (RawTLX [51]) will be used at the mid (t1, first technology set) and post-assessment (t2, second technology set).

#### Semi-structured interviews.

Before the intervention begins, participants will be interviewed to understand prior post-stroke rehabilitation experiences and expectations towards technology-aided rehabilitation. Directly after the end of the intervention, patients will take part in an interview to assess their experience and perceived benefit from this new model of care. Similarly, also therapists’ experiences and expectations will be assessed via interviews before the trial starts and after approximately one year. All interviews consist of two parts: The first part comprises explicit questions that should be rated on a Likert scale, while the second part involves qualitative, open-ended questions. The interviews will be audio-recorded.

#### HrQoL.

The perceived quality of life of the participants will be evaluated with the standardized tools EQ-5D [52] and the Stroke Specific Quality of Life Scale (SS-QoL) [53] before the intervention (t0), immediately after the intervention (t2), and during the follow-up period (t3, t4).

#### Demographics and economic data.

Besides basic demographic data such as sex and age, data on the socio-economic status as well as the housing type and location, monthly household income, and caregiver salary will be collected for the economic analysis during the baseline assessment (t0). In addition, for the cost estimation of the TARGeT program, any change in medical consumption and productivity due to this intervention will be determined with validated questionnaires of the Institute for Medical Technology Assessment Medical Consumption Questionnaire (iMCQ) [54] and Productivity Cost Questionnaire (iPCQ) [55] which will be filled in by the participants before the intervention (t0) and during the Follow-up phase (t3, t4). Furthermore, additional cost data contributing to the overall cost of the therapy such as the salary ranges of the clinical personnel, travel costs for in-clinic and home visits, costs for technical maintenance, and electricity consumption will be estimated based on the device usage time, and all therapists, technical personnel, stroke survivors, and caregivers will be asked to report the time spent on the study in journal and report forms.

### Data management and remote monitoring of technology usage

#### Data management.

Outcomes from standardized clinical assessments, usability scales, quality of life questionnaires, subject characteristics, and cost questionnaires will be collected by therapists of TTSH and electronically stored on dedicated NHG-REDcap platforms. Technology data will be automatically transferred to the secured servers at the Future Health Technologies (FHT) program, Singapore-ETH Centre, and stored there in adequate databases. Similarly, self-reported data collected with journal and report forms will be digitized and also stored on secured servers alongside the audio recordings of the interviews. No formal data monitoring committee is planned, as the study is a feasibility trial, with safety and progress overseen by the study team.

Data will be made available upon request.

#### Remote monitoring of technology usage.

**Fig 3:**
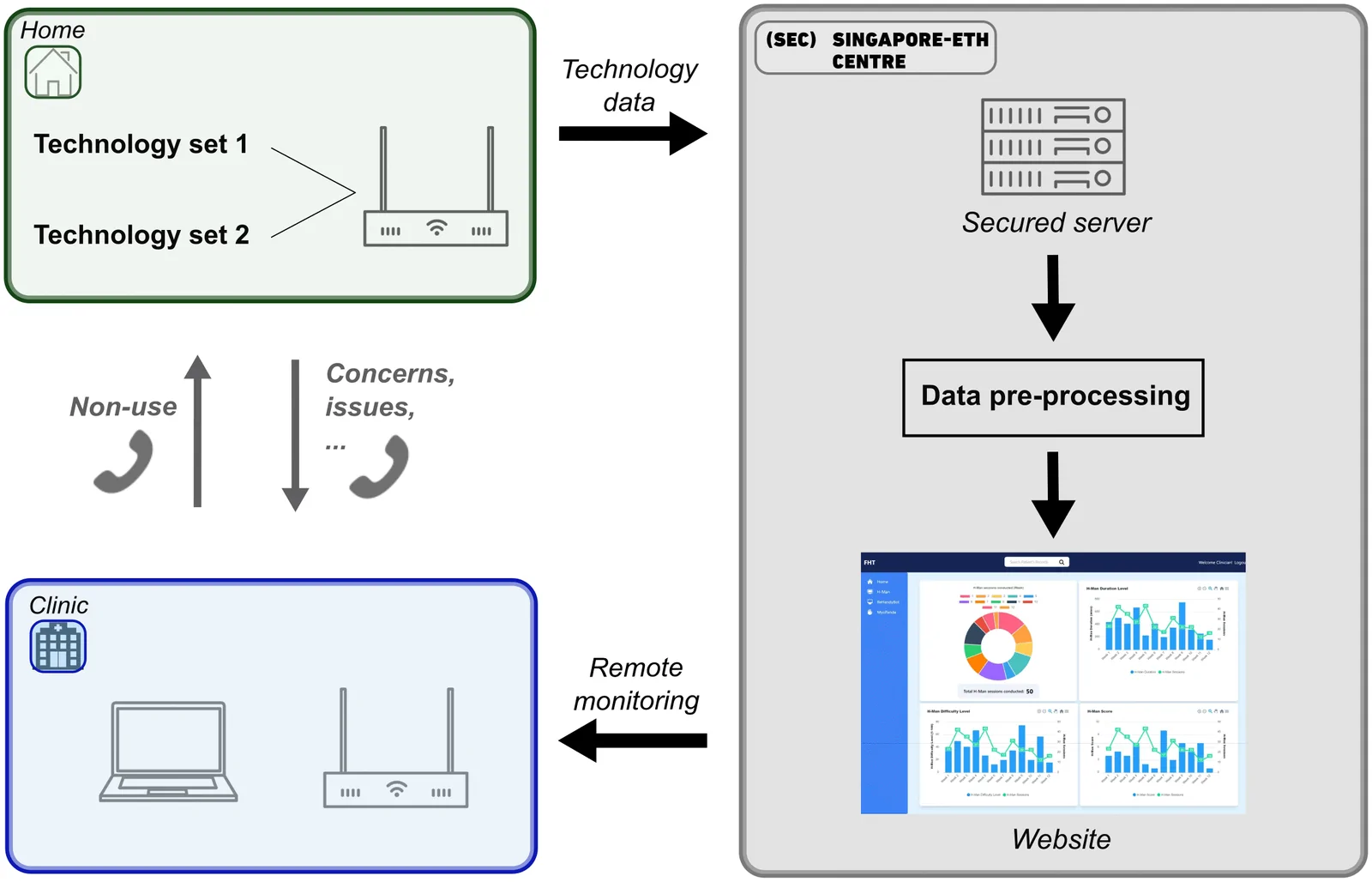
Data transfer structure. Data collected with the devices at participants’ homes will be sent to the FHT server and made available on a website allowing clinicians to remotely monitor the training progress.

To enable therapists in the clinic to remotely monitor participants’ training at home, a data transfer structure will be set up (Fig 3). Training data of the technologies will be automatically transferred from participants’ homes to the secured servers at FHT, where data will be pre-processed and made available on a website accessible to the therapists in the clinic.

For this, a 4G network will be set up alongside the technologies at a participant’s home that enables automatic syncing of the technology data to dedicated servers at FHT. All data is linked to participant-specific, anonymous identifiers. These identifiers will not be associated with the personal information of participants. On these servers, these data will be partly processed such that the attendance, the training dose with the individual technology, the different exercises executed, the movement repetitions, and the performance of the participant in the different exercises per technology will be provided to the website for remote monitoring. The performance measures align with the specific goals of the exercises and the technology and have been previously validated. For example, for a coordination-focused exercise on H-Man, the smoothness performance will be displayed. For data protection reasons, the website will be hosted on a secure server at FHT and will be publicly restricted. Therapists can access the website from the clinic after logging in with a two-factor authentication. This data will inform therapists about the participants’ training activities and compliance with the therapy plans. Additionally, the platform also allows to track the amount of time that therapists spent on the monitoring.

At home, all technologies will be employed with restricted access to only the application-specific software required for the operation of the technologies. With this restriction, the usability for participants and caregivers will be enhanced and the collected data will be protected.

### Data analysis

To evaluate the feasibility and safety of the TARGeT program for post-stroke survivors, we want to test the hypothesis that participants complied with the therapy plan and confirm that no major safety-related incidents occurred and concerns are raised. Studies exploring the unsupervised training with single devices found high adherence to the therapy plan (training on at least 80% of the days [18] and for at least 30 minutes [10,18]). We expect that training with a set of technologies at home will yield similar results. For this, we will perform descriptive analyses on the adherence to the therapy plan, i.e., we will analyze the attendance data (days trained out of six weeks) and the training dose (total time trained per day relative to therapy plan). Also, we will investigate whether financial resources (monthly household income) correlate with adherence. Moreover, we will analyze the journal and report forms of the participants, caregivers, clinicians, and technical personnel (frequency and type of concern (technical, general)), and the semi-structured interview data post-intervention by clustering according to safety and feasibility concerns.

Building on previous experiences [10,18,28], we hypothesize that the selected set of technologies is highly usable also in an unsupervised setting and that the user experience of the intervention is predominantly positive from participants and therapists. To evaluate the usability, we will analyze the SUS descriptively as well as the quantitative part of the semi-structured interviews post-intervention and perform a thematic analysis of the qualitative part of the interview. For the user experience, RawTLX and the interview data will be evaluated accordingly. Additionally, therapists’ expectations and perceived benefits will be determined by a descriptive and content analysis of the respective interview parts.

To evaluate the clinical impact of the TARGeT program, a two-way repeated measures analysis of variance (ANOVA) will be conducted for each clinical scale *time point* and *intervention group* as factors. The *time point* factor (pre, mid, post, follow-up 1, follow-up 2) is intended to assess both the immediate effects of the intervention and the long-term retention of the treatment gains. We hypothesize that motor function as assessed by ARAT etc. will improve significantly during the intervention period and that these gains will be maintained at follow-up. The *intervention group* factor (TARGeT intervention, conventional therapy) will compare the effects of the TARGeT intervention on clinical scales with duration-matched conventional therapy data from previous studies and the literature [9]. In addition, the interaction between *time point* and *intervention group* will be examined to determine whether temporal changes differ between groups. We hypothesize that the clinical impact (FMA, etc.) of the TARGeT program will be comparable to that of conventional therapy, validating the program as an effective technological alternative to traditional rehabilitative methods. Any additional health benefit in terms of quality of life will be determined by descriptive analysis of the SS-QoL and EQ-5D scales. Note that if the data violates the assumptions for parametric testing (normality – Shapiro-Wilk test, homogeneity of variance – Levene’s test), we will instead utilize the non-parametric test for these comparisons (e.g., Aligned Rank Transform ANOVA).

In addition, we will analyze whether the TARGeT program has the potential to reduce costs for health systems and society (compared to conventional therapy). For this, firstly, we will estimate the average total costs for participants of the TARGeT program. Based on their socio-demographic data, an estimate for the rent (housing type and location) as well as for the commuting costs for the in-clinic session (location) will be derived. The total cost from the societal perspective estimates of the TARGeT program will include both healthcare and non-healthcare costs. Healthcare costs will be estimated using a time-driven, activity-based costing approach, by combining costs incurred from the therapist’s (for remote monitoring, etc.) and technical personnel’s time (for device setup etc.) based on time data from journal and report forms and salary ranges, rental fees and maintenance costs for devices and furniture (based on time data from journal and report forms and salary ranges), and the participant’s healthcare service consumption during the TARGeT program (based on iMCQ data). Additionally, we will estimate non-healthcare costs, such as productivity losses borne by informal caregivers assisting participants during the TARGeT program, as well as social services consumption by the participants (based on time data from journal and report forms and iPCQ data). Furthermore, we will descriptively evaluate the impact of the therapy model on healthcare service utilisation (based on iMCQ data) and on productivity loss-related costs (based on iPCQ data). Finally, we will estimate the potential cost savings associated with the TARGeT program by comparing the costs with those of conventional therapy in a clinic, which will be derived from literature [18].

### Status and timeline of the study

Data collection started on 12 August 2024 prior to publication of this manuscript and continues until 05 June 2026. Complete analyses will be performed after the completion of data collection and the results will be shared after data analyses are finalized.

## Discussion

This protocol paper outlines a proof-of-concept and feasibility study designed to evaluate the potential of a unique home-based, multi-technology therapy concept for unsupervised technology-assisted upper-limb rehabilitation in stroke survivors. The proposed TARGeT program aims to address key challenges in current stroke neurorehabilitation, including limited access to therapy, high costs, and the difficulty of maintaining functional gains in the outpatient phase. This study will assess this therapy model’s feasibility, safety, and user experience and aims to provide preliminary insights into its potential clinical impact and economic benefits.

Despite the growing use of rehabilitation technologies in clinical settings and the advocated benefits of using technology to promote decentralized rehabilitation [16], to the best of our knowledge, this is the first study to advance home-based neurorehabilitation by offering a suite of actuated (robotic) and non-actuated technologies for unsupervised therapy directly in stroke survivors’ homes. This has the potential to address the increasing demand for neurorehabilitation while tackling the shortage in clinical manpower and reducing the traveling inconvenience by providing comprehensive, high-dose, home-based therapy. Enabling stroke survivors to perform unsupervised therapy at home allows them to train at their convenience, independent of scheduled therapy sessions at clinics or rehabilitation centers. This flexibility may enhance therapy compliance [17], increase the total therapy dose received [10,56], and improve patient satisfaction [57], while delivering clinical benefits comparable to in-clinic therapy [58,59]. This study harnesses the potential of home-based therapy by deploying complementary technologies that target both proximal and distal upper extremities and include somatosensory, motor as well as cognitive components. This unique combination provides flexibility to design a holistic therapy plan suitable for most stroke survivors, and that could efficiently address the complexity of upper limb (and in particular hand) sensorimotor function. It moreover allows to adjust the training more specifically to the changing needs along the rehabilitation journey of stroke survivors, just like in clinical practice. The technologies employed were all previously successfully tested individually, are scalable, and easy to use, such that we assume that they can be used at home in an unsupervised manner. To ensure high quality therapy at home, users will be trained on the operation of the devices in few onboarding sessions in the clinic and at home with the help of clinical personnel. In addition, therapists stay involved in the therapy by being available on demand and by remotely monitoring the training progress from the clinic. The outcomes of the study will inform on the feasibility, safety, usability and user experience and give insights into the clinical and economical comparability to conventional therapy. It will also pave the way for a future larger-scale TARGeT study to investigate the cost-effectiveness of such a novel home rehabilitation service.

Alongside the increased access to therapy, the fully unsupervised training at home has the potential to drastically reduce the demand for trained therapists. During the six weeks of daily high-intensity training at home, only two conventional therapy sessions in the clinic and five technology-onboarding sessions are scheduled. Additionally, therapists will remotely monitor the stroke survivors’ training progress via a dedicated web-based interface to ensure the quality and safety of the at-home therapy and get in contact in case of non-use of the technologies. Unlike in traditional therapy or in recent telerehabilitation trials [60,61], this progress check will be done asynchronously from the training times of the stroke survivors and, thus, gives therapists full flexibility to accommodate these checks to their schedule and downtimes, e.g., between clinic/home visits. The reduced number of clinic visits further reduces the travel expenses and burden on caregivers. On the other hand, our blended intervention approach ensures the therapy is tailored to the individual by integrating in-person and digital methods to best support the user at home and to optimize the functional gains.

By freeing up therapists’ time, this therapy model has the potential to directly decrease the direct costs of the service. Moreover, shifting the training location to the home environment lowers transportation costs. Not-scheduled, unsupervised therapy, furthermore, has the potential to reduce costs linked to productivity loss of the stroke survivor since it allows scheduling the training as per one’s convenience. Up until now, economic evaluations of technology-aided rehabilitation remain scarce [62], particularly in decentralized settings, yet such evaluations are crucial for assessing the real-world viability of these novel therapy concepts.

The comprehensive outcomes of this feasibility study will inform whether a statistically powered randomized controlled trial is feasible and necessary to fully assess the potential of the TARGeT concept. Therapists’ inputs on the technology order and the assigned therapy plans, linked with individual stroke survivor’s characteristics and performance data with the technologies, can potentially inform on the most suitable technology and exercise for a specific impairment status [16,63,64]. This can be a further step towards more autonomous therapy without supervision leading to potentially easier access to high-quality neurorehabilitation.

Despite its innovative design, this study has several limitations. First, as a proof-of-concept feasibility study without a randomized controlled design, conclusions regarding clinical effectiveness and comparability to conventional therapy will be limited and should be interpreted with caution. Second, adherence to unsupervised home-based training may vary substantially across participants and may be influenced by unmeasured factors such as motivation, or personal circumstances. And third, caregiver experience and perceived burden are not comprehensively assessed which may prevent capturing the full impact of such a therapy program.

In summary, this protocol sets the foundation for evaluating the feasibility, usability, and safety of an unsupervised, at-home multi-technology rehabilitation model. Findings from this study are expected to inform further development and refinement of the protocol and the technologies and may ultimately contribute to establishing technology-aided home rehabilitation as a viable complement to traditional therapy paradigms.

## Supporting information

S1 File SPIRIT 2025 checklist. (PDF)

S2 File Study protocol (DSRB Reference Number: 2023/00527, ECOS reference number: 2023/00527, protocol version 2). (PDF)
