# Supplementary material for: Technology-Assisted Rehabilitation Gym for at-home Training (TARGeT) for stroke survivors: A study protocol for evaluating feasibility, safety, usability, clinical benefit, and costs
Source: PLoS One. 2026 Sep 2;21(9):e0355160. doi: 10.1371/journal.pone.0355160 (PMC13537544; doi:10.1371/journal.pone.0355160)
Supplement: Supplementary file 2 [file pone.0355160.s002.pdf]

# STUDY PROTOCOL

## PROTOCOL TITLE:
**Home RehabGym: a qualitative and usability evaluation of advanced technologies for home upper limb neurorehabilitation post-stroke**

## PROTOCOL NUMBER:
DSRB Reference Number: 2023/00527

**PROTOCOL VERSION:** 2.0
**PROTOCOL DATE:** 29 July 2024


### PRINCIPAL INVESTIGATOR:
Mr Christopher Kuah, Senior Principal Occupational Therapist, Tan Tock Seng Hospital Clinic for Advanced Rehabilitation Therapeutics (TTSH CART)

### SITE PRINCIPAL INVESTIGATOR:
Dr Chua Sui Geok Karen, Senior Consultant, TTSH CART
Mrs Ng Chwee Yin, Principal Occupational Therapist, TTSH CART
Mrs Tegan Plunkett, Principal Occupational Therapist, TTSH CART
Dr Wee Seng Kwee, Senior Principal Physiotherapist, TTSH CART
Miss Megan Lau Si En, Research Assistant, Tan Tock Seng Hospital
Miss Low Ai Mei Jaclyn, Research Assistant, Tan Tock Seng Hospital
Miss Chong Wei Binh, Research Assistant, Tan Tock Seng Hospital


### STUDY SITE:
Tan Tock Seng Hospital Clinic for Advanced Rehabilitation Therapeutics (TTSH CART)


### STUDY TEAM MEMBERS:
Dr Simone Kager, Postdoctoral Researcher, Singapore-ETH Centre (SEC)
Dr Hsiao-Ju Cheng, Postdoctoral Researcher, SEC
Dr Anoop Kumar Sinha, Postdoctoral Researcher, SEC
Mr Tengiz Samkharadze, PhD student, SEC
Mr Hao Ping Lin, PhD student, SEC
Ms Irina Hungerbuehler, Engineer, SEC
Mr Chi Onn Tang, Engineer, SEC
Ms Eva Josse, PhD student, SEC
Ms Natalie Tanczak, PhD student, SEC
Mr Isaac Lu, Software Engineer, SEC
Mr Daryl Chong, Research Coordinator, SEC

# TABLE OF CONTENTS


1. BACKGROUND AND RATIONALE ..... 4
    1.1. GENERAL INTRODUCTION ..... 4
    1.2. RATIONALE AND JUSTIFICATION FOR THE STUDY ..... 5
    A. RATIONALE FOR THE STUDY PURPOSE ..... 5
    B. RATIONALE FOR STUDY POPULATION ..... 6
    C. RATIONALE FOR STUDY DESIGN ..... 6
2. HYPOTHESIS AND OBJECTIVES ..... 7
    2.1. HYPOTHESIS ..... 7
    2.4. POTENTIAL RISKS AND BENEFITS: ..... 7
    A. END POINTS - EFFICACY ..... 7
    B. END POINTS - SAFETY ..... 7
3. STUDY POPULATION ..... 8
    3.1. LIST THE NUMBER OF SUBJECTS TO BE ENROLLED. ..... 8
    3.2. CRITERIA FOR RECRUITMENT ..... 8
    3.3. INCLUSION CRITERIA ..... 8
    3.4. EXCLUSION CRITERIA ..... 9
    3.5. WITHDRAWAL CRITERIA ..... 9
    3.6. SUBJECT REPLACEMENT ..... 9
4. TRIAL SCHEDULE ..... 10
5. STUDY DESIGN ..... 12
    5.1. SUMMARY OF STUDY DESIGN ..... 12
6. METHODS AND ASSESSMENTS ..... 12
    6.1. RANDOMISATION AND BLINDING ..... 13
    6.2. CONTRACEPTION AND PREGNANCY TESTING ..... 13
    6.3. STUDY VISITS AND PROCEDURES ..... 14
7. TRIAL MATERIALS ..... 16
    7.1. TRIAL PRODUCT (S) ..... 16
    7.2. STORAGE AND DRUG ACCOUNTABILITY ..... 17
    7.3. RATIONALE FOR SELECTION OF DOSE ..... 17
8. TREATMENT ..... 17
    8.1. STUDY DRUG FORMULATIONS ..... 17
    8.2. STUDY DRUG ADMINISTRATION ..... 17
    8.3. SPECIFIC RESTRICTIONS / REQUIREMENTS ..... 18
    8.4. BLINDING ..... 18
    8.5. CONCOMITANT THERAPY ..... 18
9. SAFETY MEASUREMENTS ..... 18
    9.1. DEFINITIONS ..... 18
    9.2. COLLECTING, RECORDING AND REPORTING OF "UNANTICIPATED PROBLEMS INVOLVING RISK TO SUBJECTS OR OTHERS" – UPIRTSO EVENTS TO THE NHG DOMAIN SPECIFIC REVIEW BOARDS (DSRB) ..... 18
    9.3. COLLECTING, RECORDING AND REPORTING OF SERIOUS ADVERSE EVENTS (SAES) TO THE HEALTH SCIENCE AUTHORITY (HSA) ..... 19
    9.4. SAFETY MONITORING PLAN ..... 19
    9.5. COMPLAINT HANDLING ..... 19

**10. DATA ANALYSIS** .......... **20**
10.1. Data Quality Assurance .......... 20
10.2. Data Entry and Storage .......... 20
**11. SAMPLE SIZE AND STATISTICAL METHODS** .......... **20**
11.1. Determination of Sample Size .......... 20
11.2. Statistical and Analytical Plans .......... 20
**12. ETHICAL CONSIDERATIONS** .......... **21**
12.1. Informed Consent .......... 21
12.2. IRB review .......... 21
12.3. Confidentiality of Data and Patient Records .......... 21
**13. PUBLICATIONS** .......... **22**
**14. RETENTION OF TRIAL DOCUMENTS** .......... **22**

# STUDY PROTOCOL

## 1. BACKGROUND AND RATIONALE

### 1.1. General Introduction

Stroke is a disorder characterised by significant impairment of sensorimotor and cognitive functions and has the highest prevalence of adulthood disability in the developed world (Feigin et al., 2014). In 2010, Singapore's age-adjusted prevalence of ischaemic stroke was around 125 per 100,000 person-years and that for haemorrhagic stroke was around 44 per 100,000-person-years (Krishnamurthi et al., 2013), with 9.3% of all adults aged more than 65 years affected (Venketasubramanian et al., 2015). A third of first-time stroke survivors remains physically disabled five years after stroke. In particular, hemiparetic weakness is common after stroke (Jorgensen et al., 1999), affecting over 70% of stroke survivors (Lawrence et al., 2001; Nakayama et al., 1994) with a consequent decrease in independence and quality of life.

Stroke rehabilitation aims to maximise functional independence and improve the patient's quality of life (Venketasubramanian & Chen 2008). Greater functional independence in the patient leads to reduced caregiver burden, better patient health-related quality of life and potentially lower costs of care (Koh at al., 2015). Yet, there is evidence that, in the long-term, the functional improvements gained during early rehabilitation are partially lost (Meyer et al., 2015). This may be due to non-use of the impaired limb as a result of the decreased therapy dose (i.e., number of task repetitions or therapy time) that persons with stroke receive after discharge from the rehabilitation clinic. Growing evidence shows that increasing upper limb therapy dose for stroke in in- and out-patients settings could improve functional outcomes and prevent their long-term deterioration (Lohse et al., 2014; Ward et al., 2019). However, due to limited resources such as the low therapist to patient ratio or high costs associated to rehabilitation therapy (Zimbelman et al., 2010), providing higher therapy dose to patients after discharge is highly challenging. Additionally, functional (e.g., low mobility) and social barriers (e.g., inconvenience, need for informal care) can negatively affect the use of outpatient rehabilitation services (Lim et al., 2019). Therefore, finding new models of care is critical to meet the high demand for intensive upper limb therapy, to provide easily accessible and affordable therapy.

Upper limb rehabilitation devices are introduced as a potential solution to provide quality ensured upper limb intensive therapy and decrease therapists' workload (Harwin et al., 2006; Krebs et al., 2008; Krebs et al., 1998). Technology-aided therapy can deliver task-specific, repetitive, intensive upper limb exercises safely with comparable outcomes and positive changes to neuroplasticity with more than 10-fold increases in arm repetitions (1040 repetitions/session by MIT-MANUS upper limb robot [Krebs et al., 1998; Krebs et al., 2000]), compared with conventional therapy by therapists (60-80 repetitions/session). Another crucial benefit of technology-aided rehabilitation is its potential for therapy with little or no supervision from therapists. The largest study to date involving 770 randomized patients, found reduced-supervision robot-assisted therapy to be equally effective as enhanced upper limb therapy or usual care (Rodgers et al., 2019).

These rehabilitation devices can be set up as a 'rehabilitation gym' (RehabGym), where patients can interact with several devices that target different body and rehabilitation domains and, thus allows for a holistic and complementary therapy. Recent studies have shown that training with

such a RehabGym under reduced supervision in a hospital is not only feasible but also equally beneficial in terms of clinical outcomes (Bustamante et al., 2016; Aprile et al., 2019; Aprile et al., 2020; Hesse et al., 2014). Significant improvements in stroke recovery via telerehabilitation from motor deficits, higher cortical dysfunction and improved depression outcomes in the intervention groups, including positive self-reported functional outcomes compared to usual care during the first 3 months of stroke have been reported by several authors worldwide (Sarfo et al., 2018, AsanoM 2019, Koh 2018; Sarfo et al. 2018). Taking this even further, realizing 'on- demand' therapy by employing devices at patients' homes or in nearby community centers has the potential to create a substantial change in how care is provided. To the best of our knowledge, however, a RehabGym has never been set up at a patient's home. Therefore, in this study we aim to test the feasibility of RehabGym at home that allows for on-demand technology-aided therapy with a set of devices therewith providing complementary, unsupervised therapy that potentially decreases the costs of therapy since less clinical manpower is required and the travel costs on patient side are reduced.

## 1.2. Rationale and justification for the Study

### a. Rationale for the Study Purpose

Previous research has proven that technology-aided upper limb rehabilitation is non-inferior to conventional therapy in stroke patients (e.g., Rodgers et al., 2019; Budhota et al., 2021). It was also shown that therapy with rehabilitation devices in less supervised settings is feasible and effective. Based on this evidence, the next logical step to address the challenges of post-stroke therapy, namely providing high-dose, easily accessible, and affordable therapy, is to employ a RehabGym at patients' homes, allowing for independent, on-demand therapy (Lambercy et al., 2021).

Hence, in this study we aim to investigate the feasibility and safety of a RehabGym at home concept. The upper-limb rehabilitation technologies H-Man, ReHandyBot, and MyoGuide form a set of complementary, portable devices that are established, clinically tested, and allow for on-demand therapy. H-Man is a two-degree-of-freedom, table-top robot that provides sensorimotor training of shoulder and elbow (Campolo et al., 2014). It comes with a gamified environment that includes an intelligent algorithm to optimally challenge patients in their task execution. ReHandyBot is the portable version of ReHapticKnob a robot developed at ETH Zurich that provides neurocognitive therapy, focussing on hand opening and closing movements as well as prono-supination of the forearm (Metzger et al., 2011). Similar to H-Man, ReHandyBot/ReHapticKnob includes engaging exercises that adapt to the patient's respective needs and impairment. Both these devices have been tested extensively and successfully in clinical studies in Singapore and Europe before (Budhota et al., 2021; Ranzani et al., 2020; Ranzani et al., 2021). These two robots are complemented with a passive wrist/hand device, MyoGuide, with which patients train the hand and wrist function by controlling a virtual avatar with EMG activity measures (Lin et al., 2023).

With this set of technologies, we will set up a RehabGym at stroke patients' homes and aim to demonstrate the feasibility of a RehabGym at home by assessing the stroke patients' compliance to this novel therapy concept, the safety of unsupervised therapy at home, and the usability and user experience. This study shall also inform on the costs and affordability of this therapy concept in comparison to conventional therapy models.

## b. Rationale for Study Population

Patients with confirmed subacute to chronic stroke will be selected for study screening and consideration for eligibility. These patients have completed their acute phase of inpatient stroke care and rehabilitation at respective acute hospitals, including TTSH Rehabilitation Centre. At these stages of subacute to chronic motor recovery, patients are typically in the home environment and while still benefitting from rehabilitation. Recently, an intensive upper extremity clinic rehabilitation programme for chronic stroke survivors (median time 18 months post-stroke) with severe upper extremity disability (mean Fugl-Meyer-Motor Assessment FMA score 26/66) achieved clinically impactful and useful gains of 42% in motricity and 50% gains in motor function, which persisted for 6 months (Ward et al., 2019). Also, for the devices used in this study, clinical gains in sub-acute and chronic stroke populations were achieved in clinical trials (Budhota, et al., 2021, Ranzani, et al., 2020).

## c. Rationale for Study Design

The study design is a pilot, clinical feasibility trial to evaluate the feasibility of Home RehabGym concept through deployment of three rehabilitation devices at patients' homes: MyoGuide, H-Man and ReHandyBot. The patients will first familiarize in the clinic with the devices with initial assistance of the therapists and then take the devices home for independent, self-administered training under caregiver supervision.

During the 6 weeks training at home phase, patients will be provided with two devices at any one time: with only the MyoGuide being used by the patient for 6weeks. The H-Man and ReHandyBot will stay with the patient for 3 weeks (i.e., 21 days) each.

Hence, for instance, for patients A:

- First 3 weeks (21 days) consists of MyoGuide + H-Man
- Second 3 weeks (21 days) consists of MyoGuide + ReHandyBot

Followed by patient B:

- First 3 weeks (21 days) consists of MyoGuide + ReHandyBot
- Second 3 weeks (21 days) consists of MyoGuide + H-Man

This will allow a holistic training approach for the patients as the devices used will allow training of the upper arm, forearm and hand.

## 2. HYPOTHESIS AND OBJECTIVES

### 2.1. Hypothesis

The primary hypothesis of this study is that RehabGym at home is feasible and safe to use for stroke survivors.

### 2.2. Primary Objectives

The primary objective is to assess the feasibility and safety of RehabGym at home using the portable rehabilitation devices, H-Man, ReHandyBot, and MyoGuide. For this, compliance and intensity of self-paced exercise at home using metrics computed with data of the technologies as well as reports on safety will be assessed.

### 2.3. Secondary Objectives

The secondary objectives are (i) to collect participants' feedback in terms of usability and user experience with respect to the devices used and the overall RehabGym at home experience, (ii) to perform a cost analysis of home-based therapy, and (iii) to investigate clinical benefits of the intervention.

### 2.4. Potential Risks and benefits:

#### a. End Points - Efficacy

Direct benefits:

(1) Intensive upper limb therapy using a novel technological experience with motivating games which may make participants comply to the therapy and hence provide a high training dose
(2) Safety during training at home
(3) Information about the usability and user experience of RehabGym at home which helps improve the concept further
(4) Cost information necessary to determine the economic feasibility of a commercial implementation of RehabGym at home in the future
(5) Improvement in arm, wrist, and hand function

Indirect benefits:

(1) Reduced traveling costs and social burden related to clinic visits
(2) Potential for widespread adoption of (low-cost) technologies into community-based rehabilitation and home settings

#### b. End Points - Safety

Potential risks:

(1) Transient arm, wrist, and/or hand fatigue or muscle ache due to intensity of movement repetitions during training
(2) Computer related eye fatigue or cybersickness symptoms or transient headaches due to prolonged computer-based concentration

(3) Increase of arm, wrist, and/or hand weakness and/or muscle tone (temporarily)
(4) Potential risk of breach of confidentiality as study team will access patients' individually-identifiable information, but without any identifiers being placed in any of the devices.

Based on a previously completed trials with the devices used in this study, the interaction with the devices can be considered safe. In a randomised controlled trial with H-Man in 2018 (NHG 2014/00122, NCT02188628), 22 patients in the robotic arm interacted with H-Man (supervised), and none of these had side effects, adverse events or dropped out. H-Man is currently employed in a home-trial study (DSRB 2021/00156) to test the feasibility of unsupervised/independent training. To date,12 patients have undergone each one month of home training without any safety issues reported. Also, ReHandyBot has been previously tested in a study with 10 elderly and 2 stroke subjects without encountering adverse events related to the use of the device. Furthermore, the previous version of ReHandyBot (i.e., ReHapticKnob), was tested in two clinical studies in Europe with stroke patients, where no adverse events related to the robotic therapy occurred during over 330 hours of use. ReHandyBot does not directly cause harm and does not have side effects, and we do not expect to have medical consequences related to device deficiencies or insufficient efficacy (CIP 2016-02674 and 2020-00179). For MyoGuide, a usability study was conducted, in 2018, involving 8 stroke patients who underwent 10 days of in-clinic supervised training. No safety issues were reported during the study. There are no risks due to interaction with conventional therapy performed by subjects at the clinic (Lin et al., 2023).

# 3. STUDY POPULATION

## 3.1. List the number of subjects to be enrolled.

A total of up to 30 sub-acute and chronic stroke subjects will be enrolled.

## 3.2. Criteria for Recruitment

Patients will be recruited based on the inclusion criteria and exclusion criteria as stated below.

## 3.3. Inclusion Criteria

Subjects fulfilling all of the following inclusion criteria are eligible for the investigation:

(1) Clinical stroke (ischaemic or haemorrhagic) confirmed by admitting doctors and CT, CT angiography or MRI brain imaging
(2) Age 21 to 80 years, both males and females
(3) At least 28 days post-stroke
(4) Upper limb motor impairment of Fugl-Meyer Assessment (FMA) scale 20 to 60
(5) Montreal Cognitive Assessment (MoCA) > 21/30
(6) Ability to sit supported and continuously for 60 minutes
(7) Stable home abode with enough space to place technologies
(8) Has a carer/next of kin to supervise home-based exercises

## 3.4. Exclusion Criteria

The presence of any one of the following exclusion criteria at baseline will exclude the subject from the study:

(1) Functional impairment of the upper limb due to other pathologies
(2) Medical conditions incompatible with research participation: uncontrolled medical illnesses (hypertension or diabetes, ischaemic heart disease, congestive heart failure, bronchial asthma, severe /untreated depression, agitation, end stage renal/liver/heart/lung failure, unresolved cancers)
(3) Anticipated life expectancy of less than 6 months
(4) Pacemakers and other active implants
(5) Active seizures within 3 months
(6) Local factors potentially worsened by intensive technology-aided upper-limb therapy and computer-based training:
   - Spasticity - modified Ashworth Scale MAS > 2 of any upper limb muscle groups
   - Severe pain in affected arm - Visual Analogue Scale for pain VAS > 5/10
   - Skin wounds
(7) Cognitive impairment precluding study participation
(8) Severe visual impairment or visual neglect affecting ability to use technologies
(9) History of dementia, depression or behavioural problems
(10) Pregnant or lactating females will not be allowed to participate

## 3.5. Withdrawal Criteria

Participation in this study is entirely voluntary and participants may choose to withdraw from the study at any time without explanation but are required to inform the study team of their decision.

Participants may be withdrawn from the study in the following circumstances:

- Poor fit to the device that could potentially cause complications (e.g., strain)
- Safety concerns
- Serious adverse events related to the technology
- New medical problems unrelated to study intervention: recurrent stroke, new organ failure, new diagnoses rendering subject ineligible
- Not complying with the study procedures

The study may be stopped when one of the following occurs:

- Completion of recruitment of all intended subjects
- Permanent technological failure of all technologies
- Premature cessation of funds from grant or sponsor

## 3.6. Subject Replacement

Subjects who drop out during or after the onboarding will not be replaced. Subjects who drop out after informed consent or screening but prior to the onboarding will be replaced.

The data that has been collected until the time of withdrawal or drop out will be kept and analysed. This will enable a complete and comprehensive evaluation of the study.

## 4. TRIAL SCHEDULE

This trial will include the following sequential phases: ***Preparation***, ***Intervention***, and ***Follow-up*** (see
Figure **1**).

The ***Preparation*** phase serves to inform the patients about the study, to assess whether they are eligible for study participation according to the inclusion and exclusion criteria defined above, and to seek their consent for participation. After consent is given, the first in-clinic session will involve a baseline assessment (T0: Week 0), and first onboarding (OB1) session whereby the patient and/or caregiver will be trained on how to operate the first set of devices, MP and HM or RB. In the second onboarding (OB2) session held in-clinic, patients will slowly transit from a fully supervised operation and training mode (i.e., the research therapist instructs the patient on how to use the device and closely observes the patient while training and interferes whenever needed), to a minimally supervised training (i.e., the research therapist is only available on demand). For both onboarding sessions (OB1 and OB2), they are not considered as intervention with a therapeutic effect but serve to qualify patients and/or care givers in the independent operation and interaction with the devices.

After the in-clinic onboarding, the first 2 devices will be brought to the patients' homes at the third onboarding (OB3) session, which includes devices set-up and caregiver training. The ***Intervention*** phase then follows on for six weeks (42 days) with a change between H-Man and ReHandyBot only after three weeks (i.e.. 21 days). The MyoGuide will stay on 6 weeks. At this device swap point, at end of 3rd week, between H-Man and ReHandyBot, a fourth onboarding (OB4) session will be held for the new device in clinic plus a midway evaluation termed as T1: Week 3. This is immediately followed by a fifth onboarding (OB5) at home with new device setup and caregiver training.

Two conventional therapy sessions in the clinic (week 2 and week 5) will be provided as add-on. During the training at home phase, the study team will remotely monitor the patients' training progress once weekly and will call to check if data appears of concern, such as, missed sessions or missing data. Moreover, the patients will be provided with a phone number of the clinical study team to contact if needed during the at home training phase. At the end of this home training phase, all devices will be collected from patient's homes and patient will come into clinic for post-intervention evaluation termed as T2: Week 6.

The intervention phase will be followed by a ***Follow-up*** phase to assess long-term effects of the intervention. The first follow-up evaluation is T3: Week 12 and the second is T4: Week 24.

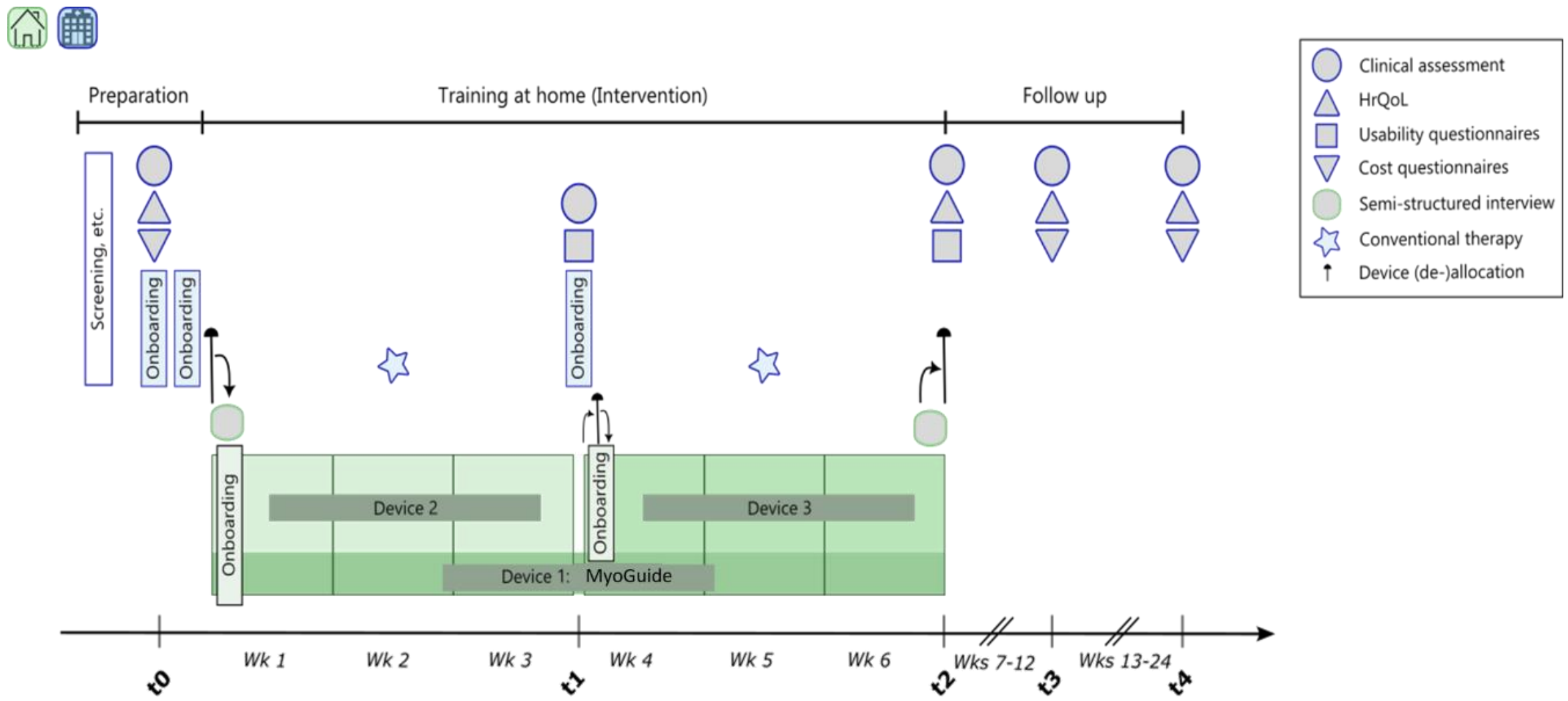


Figure 1 Trial protocol

*Phase 1: Preparation*

1. Identification of suitable subjects from inpatient discharge at TTSH rehabilitation centre and outpatient patient lists.
2. Request from patients' primary physicians for eligibility screening and contact.
3. Counselling and informed consent in rehabilitation wards at TTSH or TTSH rehabilitation specialist outpatient clinics, and enrolment and assignment of training schedule.
4. Screening for eligibility of the patient according to the inclusion and exclusion criteria.
5. Two onboarding sessions (each up to 120 minutes) with the first set of devices comprising MyoGuide (Device 1) and either H-Man or ReHandyBot (Device 2).
6. Baseline assessment T0: Week 0 at first onboarding session

*Phase 2: Intervention (Training at home)*

1. Installation and set up of first set of devices at home.
2. One onboarding session (including caregiver training) in the home of the patient with the study team (up to 120 minutes). If deemed necessary, more onboarding sessions can be arranged.
3. Home-based training for three weeks with the first set of devices and one add-on conventional therapy session in the clinic in week 2. During this training phase, the study team will remotely monitor the patients' training progress at least once a week.
4. After three weeks, mid-way assessment T1: Week 3, in the clinic and in-clinic Onboarding session (up to 120 min) with H-Man or ReHandyBot (Device 3), whichever was not part of the first set of devices.
5. One device onboarding session (including caregiver training) in the home of the patient with the study team for H-Man or ReHandyBot whichever applicable (up to 120 minutes). If deemed necessary, more onboarding sessions can be arranged.
6. Home-based training for three weeks with the second set of devices (MyoGuide and ReHandyBot or H-Man) and one add-on conventional therapy session in the clinic in week 5. During this training phase, the study team can remotely monitor the patients' training progress at least once a week.
7. After six weeks, post intervention assessment T2: Week 6.
8. End of intervention

*Phase 3: Follow-up*

1. Follow-up assessment in the clinic T3: Week 12
2. Follow-up assessment in the clinic T4: Week 24
3. End of study

# 5. STUDY DESIGN

## 5.1. Summary of Study Design

This study is designed as a pilot and feasibility study. The outcome measures serve to address the objectives of the study. These include device data metrics, reports on safety, usability and user experience assessments, cost questionnaires, semi-structured interviews, as well as clinical assessments (impairment-based and functional).
This study will help to inform about future confirmatory studies (e.g., randomized control trial to assess the efficacy of Home RehabGym).

# 6. METHODS AND ASSESSMENTS

In this study, patients will undergo a six-week at home training with rehabilitation devices. Patients will be recommended a minimal daily therapy time of 60-120mins. Before taking devices home, patients and/or their respective care givers will be trained in the clinic on how to operate and train with the devices (Onboarding). In the Onboarding sessions, patients will first interact with the devices under full supervision of a therapist before slowly transitioning to semi-supervised use, i.e. the therapist only interferes if required. This onboarding shall prepare patients and/or care givers for the independent use at home.

To achieve the objectives of this study, namely to investigate the feasibility and safety of home-based technology-aided therapy, to collect feedback on the usability and user experience, to perform a cost analysis of this therapy model and to examine clinical benefits, outcome assessments as described in the following will be conducted at baseline T0: Week 0, midway intervention phase at T1: Week 3, at end of intervention phase T2: Week 6, first follow-up at T3: Week 12, and second follow-up at T4: Week 24. Moreover, patients will be provided with a therapist's phone number to contact if needed. In addition, the study team will remotely monitor the patients' training progress, via e.g. cloud based monitoring, phone or video call, or haptic interaction, and adjust device settings as required. The study team will log any contact with the patients outside of the scheduled assessment, onboarding or therapy sessions. Video and/or audio recording may be conducted if patients consent to it. Audio-recordings are for the semi-structured interviews. For purpose of motion capture analysis, webcam recordings from H-Man during training will be captured and encrypted, and sent to a dedicated secure cloud server. Recordings will be case coded and no identifiers will be used in these audio/video recordings and any facial features will be heavily pixelated.

Therapists will also be invited to complete an interview post-study to obtain feedback on the feasibility of these devices.

Device usage and safety:

1. Device data, such as the aggregated number (mean/day and total cumulative, etc.) of task repetitions and training time (mean/day and total cumulative, etc.) per device.
2. Safety reports: Patients will be provided with a therapist's or therapist assistant's phone number to contact whenever needed, as well as the collaborator (SEC)'s number for technical support.
3. Study teams' reports on patient contact.

Usability and user experience:

4. System Usability Scale (SUS)
5. Raw Task Load Index (RawTLX)
6. Semi-structured interviews (audio-recorded) to understand patients' previous rehabilitation experience, their expectations, and the user experience of this therapy concept including patient reported outcome measures using subjective scales (Likert scale) to questions like would they prefer this to be part of standard therapy, etc. (see Appendices 1 and 2).

Health-related Quality of Life scales (HrQoL):

7. EQ-5D
8. Stroke Specific Quality of Life scale (SS-QOL)

Cost questionnaires:

9. Productive Cost Questionnaire (iPCQ)
10. Medical Consumption Questionnaire (iMCQ)

Clinical:

11. Fugl-Meyer Assessment (FMA)
12. Action Research Arm Test (ARAT)
13. Box-and-Block Test (BBT)
14. Grip strength (GS) measured by Digital Dynamometer
15. Modified Ashworth Scale (MAS)
16. Pain Visual Analogue Score (Pain)

## 6.1. Randomisation and Blinding

As this is a feasibility, open label trial, there will be no randomisation or blinding.

## 6.2. Contraception and Pregnancy Testing

Not applicable in this study. Pregnant and lactating females are not eligible for this study.

### 6.3. Study Visits and Procedures

*a. Screening Visits and Procedures*

Screening will either be done by co-investigators based on the stated inclusion and exclusion criteria listed in this document.

*b. Study Visits and Procedures*

Proposed visit and procedure schedule (see Figure **1**):

For all clinic visits after the eligibility screening (i.e., from Visit 1 onwards), patients will be compensated with $60.00 SGD. **If all study visits to clinic are completed, a participant will be given a total of $60SGD x 8 = $480 SGD.**

*Phase 1: Preparation*
**Visit 0 (Clinic):** The patient is screened to assess the eligibility for the study. Upon fulfilment of eligibility criteria and counselling, the informed consent is signed (this visit is not reimbursed by research grant).

**Visits 1 and 2 (Clinic):** The patient learns how to operate and train with the first set of devices, Device 1 (MyoGuide) and Device 2 (H-Man or ReHandyBot), under (minimal) supervision of a therapist for two in-clinic onboarding sessions (each up to 120 minutes). These sessions may also include caregiver training. During Visit 2, the baseline assessment (T0: Week 0) will be conducted including clinical assessments, HrQoL and cost questionnaires. The cost questionnaires can be taken home by the patients to be filled in. They can be sent back to the study site (TTSH CART) or alternatively be retrieved by the study team.

*Phase 2: Intervention (Training at home)*
**Visit 3 (Home):** Home installation of loaned Device 1: MyoGuide, and Device 2: H-Man or ReHandyBot, onsite set up and at-home onboarding session including caregiver training by the study team (up to 120 min). If required, more onboarding sessions at home can be arranged. During this visit, a semi-structured interview will be conducted to understand the patient's previous rehabilitation experience and expectations. A therapist will then call the patient to check on how they are coping, 2-3 days after completion of this home onboarding session.

Weeks 1 to 3 (Home): Home-based daily training for 21 days with the provided first set of devices (MyoGuide and H-Man or ReHandyBot) for recommended minimal daily training time of 60-120mins.

**Visit 4 (Clinic):** Conventional therapy in the clinic in Week 2.

**Visit 5 (Clinic):** After 21 days at-home training, mid-assessment (T1: Week 3) in the clinic comprising clinical assessments and usability questionnaires, and onboarding session for Device 3: H-Man or ReHandyBot, whichever was not part of the first set of devices (up to 120 minutes).

**Visit 6 (Home):** Collection of Device 2: H-Man or ReHandyBot, whichever is applicable, and home installation, onsite set up and onboarding session including caregiver training by the study team (up to 120 min) for the respective other Device 3 (H-Man or ReHandyBot). If required, more onboarding sessions at home can be arranged. A therapist will then call the patient to check on how they are coping, 2-3 days after completion of this home onboarding session.

<u>Weeks 4 to 6 (Home):</u> Home-based daily training for 21 days with the provided second set of devices (Device 1: MyoGuide and Device 3: ReHandyBot or H-Man) for recommended minimal daily training time of 60-120mins.

**Visit 7 (Clinic):** Conventional therapy in the clinic in Week 5.

**Visit 8 (Home):** The devices will be retrieved by the study team and a second semi-structured interview will be conducted.

**Visit 9 (Clinic):** Week 6 post assessments (T2: Week 6) including clinical assessments, usability evaluations, and HrQoL.

*Phase 3: Follow-up*
**Visit 10 (Clinic):** Week 12 (follow-up) assessments (T3: Week 12) including clinical assessments, HrQoL and cost questionnaires. The cost questionnaires can be taken home by the patients to be filled in and sent back to the study site (TTSH CART) or alternatively will be retrieved by the study team.

**Visit 11 (Clinic):** Week 24 (follow-up) assessments (T4: Week 24) including clinical assessments, HrQoL and cost questionnaires. The cost questionnaires can be taken home by the patients to be filled in and sent back to the study site (TTSH CART) or alternatively will be retrieved by the study team.

End of study.

*c. Final Study Visit*

See b) Study Visits and Procedures.

*d. Post Study Follow-up and Procedures*

See b) Study Visits and Procedures.

*e. Discontinuation Visit and Procedures*

If the patient decides to withdraw from the study for any reasons without any adverse effects, there will be no consequences. The participation in the study is voluntary. The data collected until date of withdrawal will be analysed.

If the patient is withdrawn from the study due to adverse effects, he/she will be given appropriate care under medical supervision until the symptoms of any adverse event resolve or the condition becomes stable.

# 7. TRIAL MATERIALS

## 7.1. Trial Product (s)

**H-Man**

H-Man is a portable, table-top robotic device that allows planar movements (two degrees of freedom) targeting shoulder-elbow movements. It was designed by Campolo and colleagues at Nanyang Technological University, Singapore (Campolo et al., 2014) and is characterized by a simple mechanical design and an H-shape cabled differential transmission. The unique features of H-Man are its intrinsic safety, its ease of control, owing to its homogenous workspace, low-cost and compact footprint (880mm by 700mm, 14 kg). Through a gamified environment (i.e., exergames), H-Man provides repetitive exercises needed to regain motor control and strength of the arms. It incorporates an intelligent algorithm that senses the condition of the user's arm in terms of strength and agility and automatically adjusts assistance or resistance according to the user's needs. Users interact with the H-Man to relearn sensorimotor control for resuming daily activities. H-Man has been successfully employed in post-stroke neurorehabilitation therapy and assessment of sensorimotor functions in stroke patients (Hussain et al., 2016, 2017; Cherpin et al., 2019), and is certified as a CE class 2A upper limb rehabilitation robot suitable for hospital, clinic, and home-based use in 2020 by Health Sciences Authority, Singapore (Figure 2, left).

**ReHandyBot**

ReHandyBot is the portable version of ReHapticKnob, a two degree-of-freedom robotic device designed for hand therapy and assessments post-stroke (Ranzani et al., 2020; Ranzani et al., 2021; Devittori et al., 2022) at the Rehabilitation Engineering Laboratory (RELab) of ETH Zurich (Figure 2, middle). It provides functionally relevant rehabilitative tasks that train hand opening-closing (i.e. grasping), and pronosupination of the forearm, particularly haptic perception, proprioception and motor function (Metzger et al., 2013). The exergames displayed on the interactive screen employ a neurocognitive therapy approach based on the Perfetti method (Metzger et al., 2014), and the difficulty of these exercises are adapted automatically to the user.
ReHandyBot builds on the ReHapticKnob therapy platform (Metzger et al., 2011) in that it is based on the same mechanical, software and therapy concept, and allows to perform the same therapy exercises and assessments (Metzgeri et al., 2014). To be suitable for home use, the ReHandyBot hardware (supporting structure, electronics box, etc.) was optimised and scaled down to a portable dimension while maintaining the same characteristics of the ReHapticKnob hardware.
ReHapticKnob and an initial version of ReHandyBot have been deemed feasible, safe to use and training with ReHapticKnob was proven to be non-inferior to conventional therapy in studies with stroke patients. (Ranzani et al., 2020; Ranzani et al., 2021; Devittori et al., 2022).

**MyoGuide**

MyoGuide comprises a tablet and a commercial EMG armband (Myo armband) to train wrist and hand movement control (Figure 2, right). The exergames involve an automated algorithm to detect wrist extension as well as different functional hand movements, and asks participants to repeatedly perform various functional hand/wrist movements based on specific objects.
A pilot study conducted in subacute stroke survivors in 2018 demonstrated beneficial effects on improving not only the hand/wrist impairments but also the overall upper limb impairments (full manuscript in preparation; Lin et al., 2023).

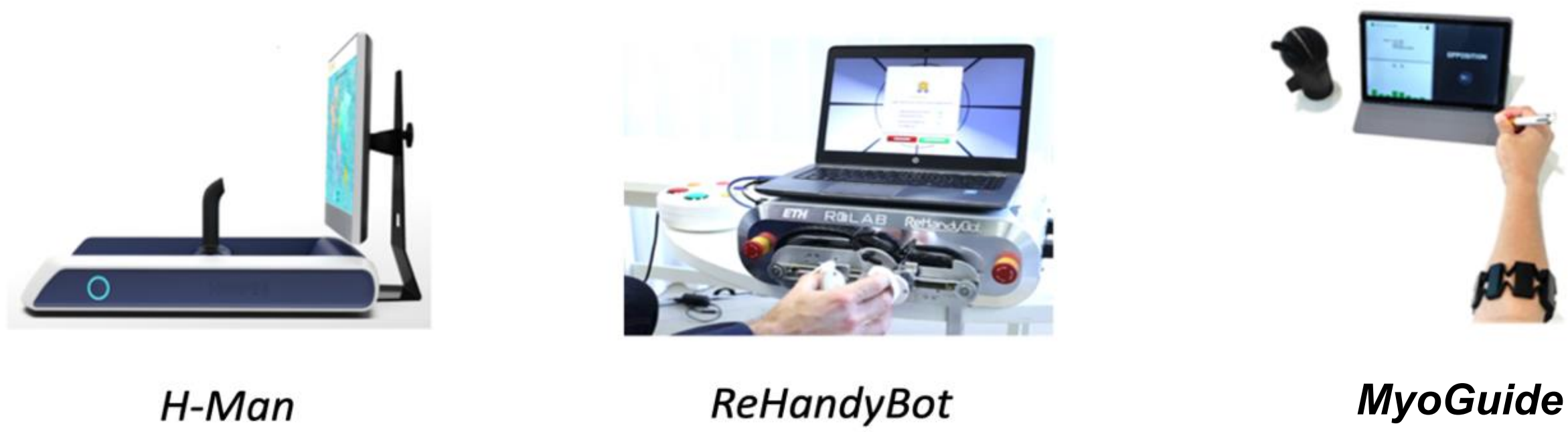


Figure 2 Devices that will be employed at patients' homes for independent training: H-Man, ReHandyBot, MyoGuide.

## 7.2. Storage and Drug Accountability

There are no special storage requirements for the devices. The devices will be stored in a secured clinic TTSH-CART. Only authorised personnel (TTSH PIs, Co-PIs & collaborators) will have access to it. Passwords unique to each individual subject will be provided to allow access for devices use during the home training.

## 7.3. Rationale for Selection of Dose

The duration of the intervention is determined by available literature on rehabilitation devices and previous concluded RCT trials, e.g. the H-Man RCT in 2018 which indicated safety and efficacy of paired conventional therapy over 6 weeks (total of 18 hours of RAT and 9 hours of paired conventional therapy over 6 weeks, NHG DSRB 2014/00122, NCT 02188628).

# 8. TREATMENT

## 8.1. Study Drug Formulations

Not applicable.

## 8.2. Study Drug Administration

Not applicable.

### 8.3. Specific Restrictions / Requirements

No restrictions or limitations on existing upper limb or lower limb therapy, no alterations or restrictions in patients' own medications, herbs, vitamins and mineral supplements while participating in the study.

### 8.4. Blinding

Not applicable.

### 8.5. Concomitant therapy

No interruptions or alterations to existing medical or rehabilitation therapies or medications or other alternative treatments will be enforced during the research trial.

## 9. SAFETY MEASUREMENTS

### 9.1. Definitions

**Deaths and life-threatening events:** Nil.
**Serious Adverse Events (SAE):** Nil.
**Other adverse event:** Nil.

### 9.2. Collecting, Recording and Reporting of "Unanticipated Problems Involving Risk to Subjects or Others" – UPIRTSO events to the NHG Domain Specific Review Boards (DSRB)

**UPIRTSO events** refers to problems, in general, to include any incident, experience, or outcome (including adverse events) that meets ALL of the following criteria:

1. **Unexpected**
   In terms of nature, severity or frequency of the problem as described in the study documentation (e.g.: Protocol, Consent documents etc).

2. **Related or possibly related to participation in the research**
   Possibly related means there is a reasonable possibility that the problem may have been caused by the procedures involved in the research; and

3. **Risk of harm**
   Suggests that the research places participants or others at a greater risk of harm (including physical, psychological, economic, or social harm) than was previously known or recognized.

**Reporting Timeline for UPIRTSO Events to the NHG DSRB.**

1. Urgent Reporting: All problems involving local deaths, whether related or not, should be reported immediately – within 24 hours after first knowledge by the NHG investigator.

2. Expedited Reporting: All other problems must be reported as soon as possible but not later than 7 calendar days after first knowledge by the NHG investigator.

## 9.3. Collecting, Recording and Reporting of Serious Adverse Events (SAEs) to the Health Science Authority (HSA)

1. **For Industry sponsored Trials**
   All SAEs will be reported to HSA according to the HSA Guidance for Industry "Safety Reporting Requirements for Clinical Drug Trials."

2. **For Principal Investigator initiated Trials**
   All SAEs that are unexpected and related to the study drug must be reported to HSA.

"A serious adverse event or serious adverse drug reaction is any untoward medical occurrence at any dose that:

- Results in death.
- Is life-threatening (immediate risk of death).
- Requires inpatient hospitalization or prolongation of existing hospitalization.
- Results in persistent or significant disability/incapacity.
- Results in congenital anomaly/birth defect.
- Is a Medically important event.

Medical and scientific judgment should be exercised in determining whether an event is an important medical event. An important medical event may not be immediately life threatening and/or result in death or hospitalization. However, if it is determined that the event may jeopardize the subject and/or may require intervention to prevent one of the other adverse event outcomes, the important medical event should be reported as serious."

All SAEs that are unexpected and related to the study drug will be reported. The investigator is responsible for informing HSA no later than 15 calendar days after first knowledge that the case qualifies for expedited reporting. Follow-information will be actively sought and submitted as it becomes available. For fatal or life-threatening cases, HSA will be notified as soon as possible but no later than 7 calendar days after first knowledge that a case qualifies, followed by a complete report within 8 additional calendar days.

## 9.4. Safety Monitoring Plan

Data and subject feedback will be reviewed quarterly. In addition, any negative feedback in terms of subject feedback of therapist opinion will be discussed ad hoc.

## 9.5. Complaint Handling

All the complaints arising from the study will be recorded and reported to the principal or site investigator, co-investigator and the study team. Major complaints will be handled in accordance with hospital standard operating procedures and reported to NHG DSRB via email.

## 10. DATA ANALYSIS

### 10.1. Data Quality Assurance

Data will be reviewed quarterly by study team during the clinical phase of the study.

### 10.2. Data Entry and Storage

Data will be stored both on paper and electronically. The electronic data will be stored on the local drive of a networked internet-disabled password protected PC in the secured access offices of the TTSH investigators. Hard copies of research data will be stored under lock and the key in access restricted offices of TTSH researchers. Electronic data will be stored on NHG Redcap platform accessed according to varying access levels only by TTSH PI and Co I and SC.
Pseudonymized data will be stored on a secure data storage server that is located in Singapore and approved for such purposes by the TTSH IT department. Data will be kept 7 years for hard copies and 10 years for soft copies, after study completion. Data access is granted for project members based on the Research Collaboration Agreement between NHG, SEC, ETH, and NTU.

## 11. SAMPLE SIZE AND STATISTICAL METHODS

### 11.1. Determination of Sample Size

In this feasibility trial, up to 30 patients shall be recruited. Isaac and Michael (1995) have recommended the sample size for pilot studies to range between 10 to 30 and therefore we decided to go with the mid-to-high-range sample size of 25. Factoring in a ~20% drop out rate (~5 subjects), the total sample size is then 30.

### 11.2. Statistical and Analytical Plans

As the primary objective of the study concerns the feasibility and safety of home-based technology-aided therapy, no statistical analyses will be performed.

Only for the secondary objective that aims to assess the clinical benefits of Home RehabGym, the reduction of upper limb impairment of stroke patients after home-based technology-aided therapy compared with baseline will be investigated and compared with retrospective results from an earlier clinical trial concluded in 2018 (NHG 2014/00122, NCT 02188628) of similarly affected stroke patients undergoing only conventional therapy and/or data available in literature. Paired t-tests will be performed for the sample to compare changes in outcome measures between baseline and post-training and at follow-ups of the study period. The analysis of variance will be conducted to examine changes in different clinical scales with repeated measures.

## 12. ETHICAL CONSIDERATIONS

### 12.1. Informed Consent

Informed Consent will be obtained from eligible participants or their legally appointed representatives after screening and prior to any study-related procedures. The participant will be given sufficient time to decide whether or not to participate in the research. If potential participants choose not to participate in the research, this will be respected and they will be reassured that their treatment will not be affected by this decision. Only study team members who have been delegated by the PI can obtain informed consent from the participants. This will be documented in the Study Responsibility Log.

Other project members (not TTSH staff) will not have access to the participant's consent form or personal information.

### 12.2. IRB review

This protocol is submitted together with the attached informed consent documents to NHG DSRB. Given that this study will be performed as part of a collaborative research project with ETH Zurich (Zurich, Switzerland), this study protocol will also be submitted to the institutional review board of ETH Zurich.

### 12.3. Confidentiality of Data and Patient Records

The data management and privacy rules were defined to adhere to the Singaporean Personal Data Protection Act as well as the Swiss Data Protection Laws.
Data and patient records that are collected by TTSH staff will be stored both on paper and electronically. Pseudonymised codes will be used for such data and participant identifiers will be held only by clinical study team in hard copies or corporate computers, who have been delegated and trained by site and principal PIs. Electronic data will be stored in password protected desktops or laptops in the locked offices of the TTSH investigators. Hard copies of research data will be stored in a locked cabinet and the key kept in an access restricted office. Electronic data will be stored on NHG Redcap platform accessed according to varying access levels only by TTSH PI and Co I and SC.
For research collaborators (for example, SEC staff) will only deal with pseudonymised data, devoid of names, NRIC, dates of birth, dates of stroke.
In case study team members (non-clinical) need to deal with personal data (e.g., address, postal code, telephone or name of patient) for device delivery, mailing, trouble-shooting, or maintenance, such sensitive data is kept separate from the network/internet on a password protected, encrypted, and certified storage medium and immediately destroyed after the study completion by the study team (non-clinical).

**File sharing:**
1) Data will only be shared in pseudonymised form and shall be used only for the authorised purpose as stated in the Research Project.
2) Video, if consented by patient, can be shared with delegated study team without patient identifiers.

3) Data will be shared via the FHT data infrastructure SECure. Data access is granted to individuals, after they have read, understood and signed the Acceptable User Policy of SECure. Access is monitored and access rights can be modified as delegated by responsible PIs.
4) The overarching vision of the project is that access to pseudonymized or anonymized data will be granted to the project team members of the PIs from SEC, ETH, TTSH, or NTU.
5) Selected data modalities may also be shared in pseudonymized or anonymized form with researchers from other FHT modules. These researchers will get access via the FHT data infrastructure SECure.
6) Only anonymized or aggregated data will be shared with the general public. Minimal requirements are (i) participant gave consent for sharing the data; and (ii) approval from the PIs who were responsible for data collection.

## 13. PUBLICATIONS

These will be jointly prepared and written by the study team. No identifiable information of participants will be shown in any publications.

## 14. RETENTION OF TRIAL DOCUMENTS

All electronic and hard copy research data will be stored for analysis and publication for 6 years after the end of the study period. Data will thereafter be deleted.

# List of Attachments

Kindly refer to the DSRB application with attachments:

- **(i)** Informed Consent Form
- **(ii)** Screening Form
- **(iii)** Case Record Form

# References


Aprile, I., Cruciani, A., Germanotta, M., Gower, V., Pecchioli, C., Cattaneo, D., ... & Gramatica, F. (2019). Upper limb robotics in rehabilitation: an approach to select the devices, based on rehabilitation aims, and their evaluation in a feasibility study. *Applied Sciences*, *9*(18), 3920.

Aprile, I., Germanotta, M., Cruciani, A., Loreti, S., Pecchioli, C., Cecchi, F., ... & Carrozza, M. C. (2020). Upper limb robotic rehabilitation after stroke: a multicenter, randomized clinical trial. *Journal of Neurologic Physical Therapy*, *44*(1), 3-14.

Budhota, A., Chua, K. S., Hussain, A., Kager, S., Cherpin, A., Contu, S., ... & Campolo, D. (2021). Robotic assisted upper limb training post stroke: a randomized control trial using combinatory approach toward reducing workforce demands. *Frontiers in Neurology*, *12*, 622014.

Bustamante Valles, K., Montes, S., Madrigal, M. D. J., Burciaga, A., Martínez, M. E., & Johnson, M. J. (2016). Technology-assisted stroke rehabilitation in Mexico: a pilot randomized trial comparing traditional therapy to circuit training in a robot/technology-assisted therapy gym. *Journal of Neuroengineering and Rehabilitation*, *13*, 1-15.

Campolo, D., Tommasino, P., Gamage, K., Klein, J., Hughes, C. M., & Masia, L. (2014). H-Man: A planar, H-shape cabled differential robotic manipulandum for experiments on human motor control. *Journal of Neuroscience Methods*, *235*, 285-297.

Cherpin, A., Kager, S., Budhota, A., Contu, S., Vishwanath, D., Kuah, C. W., ... & Campolo, D. (2019, June). A preliminary study on the relationship between proprioceptive deficits and motor functions in chronic stroke patients. In *2019 IEEE 16th International Conference on Rehabilitation Robotics (ICORR)* (pp. 465-470). IEEE.

Devittori, G., Ranzani, R., Dinacci, D., Romiti, D., Califfi, A., Petrillo, C., ... & Lambercy, O. (2022, July). Automatic and personalized adaptation of therapy parameters for unsupervised robot-assisted rehabilitation: A pilot evaluation. In *2022 International Conference on Rehabilitation Robotics (ICORR)* (pp. 1-6). IEEE.

Feigin, V. L., Forouzanfar, M. H., Krishnamurthi, R., Mensah, G. A., Connor, M., Bennett, D. A., ... & Murray, C. (2014). Global and regional burden of stroke during 1990–2010: findings from the Global Burden of Disease Study 2010. *The Lancet*, *383*(9913), 245-255.

Harwin, W. S., Patton, J. L., & Edgerton, V. R. (2006). Challenges and opportunities for robot-mediated neurorehabilitation. *Proceedings of the IEEE*, *94*(9), 1717-1726.

Hesse, S., Heß, A., Werner C, C., Kabbert, N., & Buschfort, R. (2014). Effect on arm function and cost of robot-assisted group therapy in subacute patients with stroke and a moderately to severely affected arm: a randomized controlled trial. *Clinical Rehabilitation*, *28*(7), 637-647.

Hussain, A., Budhota, A., Hughes, C. M. L., Dailey, W. D., Vishwanath, D. A., Kuah, C. W., ... & Campolo, D. (2016). Self-paced reaching after stroke: a quantitative assessment of longitudinal and directional sensitivity using the H-man planar robot for upper limb neurorehabilitation. *Frontiers in Neuroscience*, *10*, 477.

Hussain, A., Budhota, A., Contu, S., Kager, S., Vishwanath, D. A., Kuah, C. W., ... & Campolo, D. (2017, July). Quantitative assessment of motor functions post-stroke: Responsiveness of upper-extremity robotic

measures and its task dependence. In *2017 International Conference on Rehabilitation Robotics (ICORR)* (pp. 1037-1042). IEEE.

Isaac, S., & Michael, W. B. (1995). Handbook in research and evaluation: A collection of principles, methods, and strategies useful in the planning, design, and evaluation of studies in education and the behavioral sciences. *Edits publishers*.

Jorgensen, H. S., Nakayama, H., Raaschou, H. O., & Olsen, T. S. (1999). Stroke: neurologic and functional recovery the Copenhagen Stroke Study. *Physical Medicine and Rehabilitation Clinics of North America*, *10*(4), 887-906.

Koh, G. C. H., Yen, S. C., Tay, A., Cheong, A., Ng, Y. S., De Silva, D. A., ... & Hoenig, H. (2015). Singapore Tele-technology Aided Rehabilitation in Stroke (STARS) trial: protocol of a randomized clinical trial on tele-rehabilitation for stroke patients. *BMC neurology*, *15*(1), 1-14.

Krebs, H. I., Dipietro, L., Levy-Tzedek, S., Fasoli, S. E., Rykman-Berland, A., Zipse, J., ... & Hogan, N. (2008). A paradigm shift for rehabilitation robotics. *IEEE Engineering in Medicine and Biology Magazine*, *27*(4), 61-70.

Krebs, H. I., Hogan, N., Aisen, M. L., & Volpe, B. T. (1998). Robot-aided neurorehabilitation. *IEEE Transactions on Rehabilitation Engineering*, *6*(1), 75-87.

Krebs, H. I., Volpe, B. T., Aisen, M. L., & Hogan, N. (2000). Increasing productivity and quality of care: Robot-aided neuro-rehabilitation. *Journal of Rehabilitation Research and Development*, *37*(6), 639-652.

Krishnamurthi, R. V., Feigin, V. L., Forouzanfar, M. H., Mensah, G. A., Connor, M., Bennett, D. A., ... & Murray, C. (2013). Global and regional burden of first-ever ischaemic and haemorrhagic stroke during 1990–2010: findings from the Global Burden of Disease Study 2010. *The Lancet Global Health*, *1*(5), e259-e281.

Lambercy, O., Lehner, R., Chua, K., Wee, S. K., Rajeswaran, D. K., Kuah, C. W. K., ... & Gassert, R. (2021). Neurorehabilitation from a distance: can intelligent technology support decentralized access to quality therapy?. *Frontiers in Robotics and AI*, *8*, 612415.

Lawrence, E. S., Coshall, C., Dundas, R., Stewart, J., Rudd, A. G., Howard, R., & Wolfe, C. D. (2001). Estimates of the prevalence of acute stroke impairments and disability in a multiethnic population. *Stroke*, *32*(6), 1279-1284.

Lim, M. J. R., Wong, B. T. Y., Koh, P. P. W., Raszewski, R., & Asano, M. (2019). A brief review of post-stroke employment in Singapore. *Int J Neurorehabil*, *6*(4), 354.

Lin, H. P., Zhang, X., Xu, Y., Woolley, D., Zhao, L., Liang, W., ... & Wenderoth, N. (2023). A usability study on mobile EMG-guided neurofeedback training for individuals with stroke–MyoGuide. *Archives of Physical Medicine and Rehabilitation*, *104*(3), e9.

Lohse, K. R., Lang, C. E., & Boyd, L. A. (2014). Is more better? Using metadata to explore dose–response relationships in stroke rehabilitation. *Stroke*, *45*(7), 2053-2058.

Metzger, J. C., Lambercy, O., Califfi, A., Conti, F. M., & Gassert, R. (2013). Neurocognitive robot-assisted therapy of hand function. *IEEE Transactions on Haptics*, *7*(2), 140-149.

Metzger, J. C., Lambercy, O., Califfi, A., Dinacci, D., Petrillo, C., Rossi, P., ... & Gassert, R. (2014). Assessment-driven selection and adaptation of exercise difficulty in robot-assisted therapy: a pilot study with a hand rehabilitation robot. *Journal of Neuroengineering and Rehabilitation*, *11*(1), 1-14.

Metzger, J. C., Lambercy, O., Chapuis, D., & Gassert, R. (2011). Design and characterization of the ReHapticKnob, a robot for assessment and therapy of hand function. In *2011 IEEE/RSJ International Conference on Intelligent Robots and Systems* (pp. 3074-3080). IEEE.

Meyer, S., Verheyden, G., Brinkmann, N., Dejaeger, E., De Weerdt, W., Feys, H., ... & De Wit, L. (2015). Functional and motor outcome 5 years after stroke is equivalent to outcome at 2 months: follow-up of the collaborative evaluation of rehabilitation in stroke across Europe. *Stroke*, *46*(6), 1613-1619.

Nakayama, H., Jorgensen, H. S., Raaschou, H. O., & Olsen, T. S. (1994). The influence of age on stroke outcome. The Copenhagen Stroke Study. *Stroke*, *25*(4), 808-813.

Ranzani, R., Lambercy, O., Metzger, J. C., Califfi, A., Regazzi, S., Dinacci, D., ... & Gassert, R. (2020). Neurocognitive robot-assisted rehabilitation of hand function: a randomized control trial on motor recovery in subacute stroke. *Journal of Neuroengineering and Rehabilitation*, *17*, 1-13.

Ranzani, R., Eicher, L., Viggiano, F., Engelbrecht, B., Held, J. P., Lambercy, O., & Gassert, R. (2021). Towards a platform for robot-assisted minimally-supervised therapy of hand function: design and pilot usability evaluation. *Frontiers in Bioengineering and Biotechnology*, *9*, 652380.

Rodgers, H., Bosomworth, H., Krebs, H. I., van Wijck, F., Howel, D., Wilson, N., ... & Shaw, L. (2019). Robot assisted training for the upper limb after stroke (RATULS): a multicentre randomised controlled trial. *The Lancet*, *394*(10192), 51-62.

Venketasubramanian, N., Chang, H. M., Chan, B. P. L., Young, S. H., Kong, K. H., Tang, K. F., ... & Singapore Stroke Registry. (2015). Countrywide stroke incidence, subtypes, management and outcome in a multiethnic Asian population: the Singapore Stroke Registry—methodology. *International Journal of Stroke*, *10*(5), 767-769.

Venketasubramanian, N., & Chen, C. L. H. (2008). Burden of stroke in Singapore. *International Journal of Stroke*, *3*(1), 51-54.

Ward, N. S., Brander, F., & Kelly, K. (2019). Intensive upper limb neurorehabilitation in chronic stroke: outcomes from the Queen Square programme. *Journal of Neurology, Neurosurgery & Psychiatry*, *90*(5), 498-506.

Zimbelman, J. L., Juraschek, S. P., Zhang, X., & Lin, V. W. H. (2010). Physical therapy workforce in the United States: forecasting nationwide shortages. *PM&R*, *2*(11), 1021-1029.
